# Core circadian clock and light signaling genes brought into genetic linkage across the green lineage

**DOI:** 10.1093/plphys/kiac276

**Published:** 2022-06-08

**Authors:** Todd P Michael

**Affiliations:** The Plant Molecular and Cellular Biology Laboratory, The Salk Institute for Biological Studies, La Jolla, California 92037, USA

## Abstract

The circadian clock is conserved at both the level of transcriptional networks as well as core genes in plants, ensuring that biological processes are phased to the correct time of day. In the model plant Arabidopsis (*Arabidopsis thaliana*), the core circadian *SHAQKYF-type-MYB* (*sMYB*) genes *CIRCADIAN CLOCK ASSOCIATED 1* (*CCA1*) and *REVEILLE* (*RVE4*) show genetic linkage with *PSEUDO-RESPONSE REGULATOR 9* (*PRR9*) and *PRR7*, respectively. Leveraging chromosome-resolved plant genomes and syntenic ortholog analysis enabled tracing this genetic linkage back to *Amborella trichopoda*, a sister lineage to the angiosperm, and identifying an additional evolutionarily conserved genetic linkage in light signaling genes. The *LHY/CCA1–PRR5/9*, *RVE4/8–PRR3/7*, and *PIF3–PHYA* genetic linkages emerged in the bryophyte lineage and progressively moved within several genes of each other across an array of angiosperm families representing distinct whole-genome duplication and fractionation events. Soybean (*Glycine max*) maintained all but two genetic linkages, and expression analysis revealed the *PIF3–PHYA* linkage overlapping with the E4 maturity group locus was the only pair to robustly cycle with an evening phase, in contrast to the *sMYB–PRR* morning and midday phase. While most monocots maintain the genetic linkages, they have been lost in the economically important grasses (Poaceae), such as maize (*Zea mays*), where the genes have been fractionated to separate chromosomes and presence/absence variation results in the segregation of *PRR7* paralogs across heterotic groups. The environmental robustness model is put forward, suggesting that evolutionarily conserved genetic linkages ensure superior microhabitat pollinator synchrony, while wide-hybrids or unlinking the genes, as seen in the grasses, result in heterosis, adaptation, and colonization of new ecological niches.

## Teaser

The genetic linkage of the core circadian clock and light signaling genes coincides with the rise to dominance of flowering plants and may explain environment-specific growth as well as heterosis.

## Introduction

The plant circadian clock ensures biological processes occur at the proper time-of-day (TOD) regardless of predictable as well as random environmental changes, which is why it has been a target for plant domestication (Bendix et al., 2015; Creux and Harmer, 2019; McClung, 2021; Steed et al., 2021). The molecular mechanisms of the plant circadian clock were initially worked out in the model plant Arabidopsis (*Arabidopsis thaliana*) and now there is a growing body of work across an array of crop, ornamental, and nonmodel plants (Sanchez and Kay, 2016; McClung, 2019). The circadian (*circa diem*) clock is so named because the period is approximately a day (24 h) varying between 22 and 27 h across different Arabidopsis accessions, which correlates with the latitude of origin (Michael et al., 2003). The almost 24-h period of the circadian clock enables plants to anticipate changes in photoperiod each day over the growing season, synchronizing timing of biological processes, and ultimately enhancing plant fitness (Green et al., 2002; Dodd et al., 2005). Fundamental processes in plants are under the regulation of the circadian clock such as phytohormone-regulated growth, disease resistance, and the cell cycle to highlight a few (Michael et al., 2008a; Wang et al., 2011; Fung-Uceda et al., 2018). While the specific molecular structure of the clock may vary across the green lineage (Archaeplastida), many features, including gene content and expression patterns, are conserved (Ferrari et al., 2019).

Autotrophic organisms that rely on photosynthesis for energy, from algae to plants, are dependent on synchronizing their biology to the daily changes in light and temperature. For instance, almost all of the genes in the single-celled pico-algae *Ostreococcus* show oscillations in gene expression in a TOD fashion (Monnier et al., 2010; Thommen et al., 2012), while 80% of genes in the model macroalga *Chlamydomonas reinhardtii* have TOD peak abundance (Zones et al., 2015). In most plants, over 30% of genes cycle under diurnal conditions of light and temperature, while 10%–20% cycle under circadian conditions of continuous light and temperature (Michael et al., 2008b; Khan et al., 2010; Filichkin et al., 2011; Sato et al., 2013; Nose and Watanabe, 2014; Cronn et al., 2017; Ferrari et al., 2019; MacKinnon et al., 2019; Wai et al., 2019; Greenham et al., 2020; Lai et al., 2020; Michael et al., 2020; Wickell et al., 2021). However, there is evidence that the majority of genes in plants may have TOD expression potential since in Arabidopsis 90% of all genes have TOD expression in at least one of an array of conditions tested (Michael et al., 2008b). Now there is an emerging body of literature concerning the circadian clocks across an array of plants and how they respond to natural conditions of light and temperature (Panter et al., 2019).

The molecular architecture and genes involved with the plant circadian clock were worked out in Arabidopsis, which revealed a highly complex network of negative and positive feedback loops with at least 61 genes (Lou et al., 2012; McClung, 2019). At the core of these feedback loops are the *SHAQKYF-type-MYB* (*sMYB*) sub-family of myeloblastosis (MYB) transcription factors (TFs) and *PSEUDO RESPONSE REGULATOR* (*PRR*) genes (McClung, 2019). The first two *sMYB* genes described were *LATE ELONGATED HYPOCOTYL* (*LHY*) and *CIRCADIAN CLOCK ASSOCIATED 1* (*CCA1*), both of which have peak expression at dawn (Supplemental Figure S1; Schaffer et al., 1998; Wang and Tobin, 1998). The *sMYB* sub-family also includes eight *REVEILLE* (*RVE*) genes so called because they also have peak expression at dawn (Chaudhury et al., 1999). The *RVEs* form two subclades that include *RVE1*/*2*/*7* and *RVE3*/*4*/*5*/*6*/*8*, the latter of which share an additional LHY*–*CCA1-like (LCL) domain resulting in their alternative names *LCL3/1/4/2/5*, respectively, (Rawat et al., 2009; Farinas and Mas, 2011; Gray et al., 2017). The *PRR* family is defined by conserved CCT (CONSTANS, CO-like, and TOC1) and REC (receiver) domains, and includes five genes *PRR1*/*3*/*5*/*7*/*9* that display “circadian waves of expression” at dawn (*PRR9*), midday (*PRR7*), evening (*PRR3* and *PRR5*), and early night (*PRR1*; Supplemental Figure S1; Matsushika et al., 2000). *PRR1*, also known as *TOC1* (*TIMING OF CAB 1*), was the first plant clock gene identified (Millar et al., 1995; Somers et al., 1998), and along with *CCA1* was shown to form the primary negative feedback loop (Alabadı’ et al., 2002).

**Figure 1:**
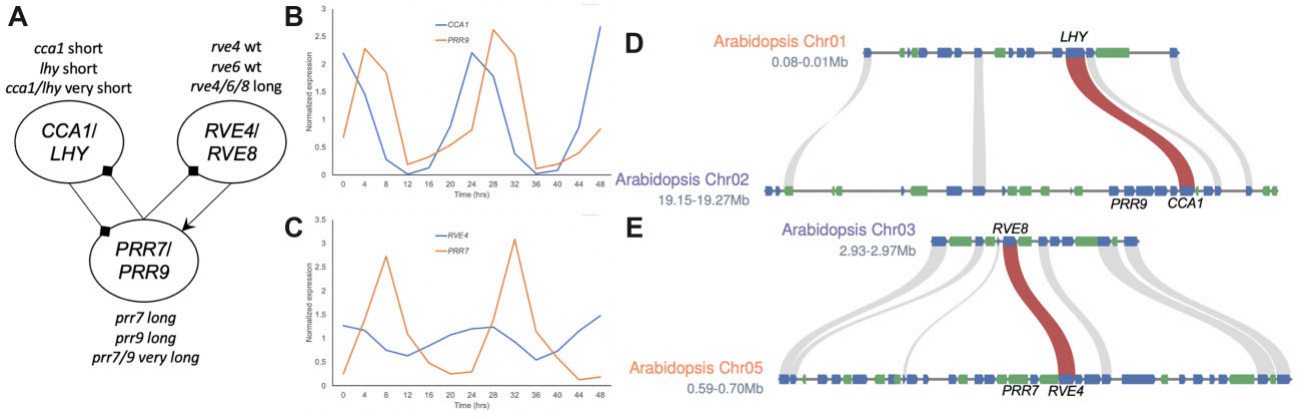
Core circadian clock genes in Arabidopsis are found in tight linkage. A, Simplified plant circadian clock model that includes the *SHAQKYF-type-MYB* (sMYB) paralogs *CCA1*, *LHY*, *RVE4*, and *RVE8*, and the *PRRs PRR7* and *PRR9*. Paralogs *CCA1/LHY* and *RVE4/8* are expressed in the morning with the former negatively regulating and the latter positively regulating the midday expressed *PRR7/9* paralogs. The circadian phenotype of the knockout mutants is included: long, long period (>24 h); short, short period (<24 h). B, *CCA1* (blue) and *PRR9* (orange) display morning (Zeitgeber Time 0; ZT0) and midday (ZT4) peak TOD expression, respectively. C, *RVE4* (blue) and *PRR7* (orange) display morning (ZT4) and midday (ZT7) peak TOD expression, respectively. D, Syntenic regions on Arabidopsis chromosome 1 (Chr01; orange) containing *LHY* and Chr02 (purple) containing *CCA1*. *PRR9* is found three genes away from *CCA1*. E, Syntenic regions on Arabidopsis Chr03 (purple) containing *RVE8* and Chr05 (orange) containing *RVE4*. *PRR7* is found two genes away from *RVE4*. The other syntenic genes (forward, blue; reverse, green) are depicted in the homologous chromosomal regions (gray).

In general, a gene is considered a core circadian clock component when its loss or gain of function results in a change in period under continuous light and temperature, or free-run conditions. For instance, the loss of either *LHY* or *CCA1* results in a short free-running period (FRP) of 21 h, while the double mutant *cca1/lhy* results in an very short FRP of 18 h suggesting that these paralogs are redundant in core circadian clock function (Figure 1A; Supplemental Note S1; Green and Tobin, 1999; Alabadı’ et al., 2002; Mizoguchi et al., 2002; Oda et al., 2007; Lu et al., 2009; Salomé et al., 2010). Of the *RVE* family, only the single loss of *RVE8/LCL5* results in a longer FRP (Farinas and Mas, 2011; Rawat et al., 2011), while the doubles (*rve4/8*, 27 h; *rve6/8*, 26 h) triple (*rve4/6/8*, 28 h), or quadruple (*rve3/4/5/6/8*, 28 h) results in a progressively longer FRP consistent with the other *RVEs* also playing a role in the core circadian clock (Hsu et al., 2013; Gray et al., 2017). Consistent with their opposite impacts on FRP, the loss of both *LHY/CCA1* and *RVE4/6/8* (*lhy/cca1/rve4/6/8* quintuple) have an essentially wild-type 24-h FRP, suggesting the two clades of *sMYB* have reciprocal and dispensable roles in maintaining FRP (Shalit-Kaneh et al., 2018). In contrast, all of the single *PRR* loss of function mutants result in changes in FRP with the evening expressed *PRRs* (*PRR1/3/5*) resulting in shorter FRP, and the morning expressed *PRRs* (*PRR7/9*) resulting in longer FRP (Michael et al., 2003; Salomé and McClung, 2005). A double mutant of the morning expressed *PRRs* (*prr7/9*) results in a very long FRP, suggesting that these *PRRs* have overlapping but distinct roles in the circadian clock (Farré et al., 2005; Salomé and McClung, 2005). However, a quintuple with *CCA1/LHY* (*cca1//lhy/prr7/9*) results in a FRP similar to *lhy/cca1* double, consistent with *CCA1*/*LHY* being epistatic to *PRR7*/*9* impacts on FRP (Salomé et al., 2010). A highly simplified model of these negative and positive feedback loops has *CCA1/LHY* negatively regulating the *PRRs*, which in turn negatively regulate both *CCA1/LHY* and *RVEs*, while the *RVEs* play a positive role in regulating the *PRRs* (Figure 1A; Supplemental Note S1; Shalit-Kaneh et al., 2018).

The circadian clock is also conserved from algae to flowering plants at both the level of the proteins as well as TOD networks (Filichkin et al., 2011; Reyes et al., 2017). While plants have more components, this core negative feedback circadian clock is conserved as far back as *Ostreococcus*, whose oscillator is made up of one *sMYB–PRR* loop (Corellou et al., 2009; Monnier et al., 2010; Morant et al., 2010). Subsequently, several studies have explored the evolution and conservation of the core circadian clock components in bryophytes, lycophytes, and a diverse array of angiosperms finding that some genes like the *sMYB* and *PRRs* are found across the green linkage, yet others have only emerged in flowering plants (Holm et al., 2010; McClung, 2010; Takata et al., 2010; Satbhai et al., 2011; Ryo et al., 2016; Linde et al., 2017; Wickell et al., 2021). However, as more nonmodel plants from distinct branches of the green lineage are analyzed, novelty in the circadian clock may become more prevalent. For instance, in the lycophyte *Isoetes taiwanensis*, which leverages TOD-specific underwater crassulacean acid metabolism (CAM) photosynthesis, the core clock components *TOC1/PRR1* and *GIGANTEA* (*GI*) are found in multiple copies with unique TOD expression (Wickell et al., 2021). While in general the core clock components are conserved in the green lineage, it is not clear how they have been inherited over evolutionary time, which is now possible to test with the availability of so many high-quality genomes and new analytical tools (Zhao and Schranz, 2019; Zhao et al., 2021).

The evolutionary history of genes across the green lineage is characterized by rounds of whole-genome duplication (WGD) and polyploidy, followed by neofunctionalization, subfunctionalization, or complete removal and fractionation of paralogous genes to ensure gene dosage balance (Cheng et al., 2018). It has been hypothesized that the enigmatic rise to dominance of the angiosperms in the Cretaceous in part resulted from long stretches of asexual polyploid hybrid vigor followed by a return to sexual diploids with broad phenotypic and species diversity (Soltis et al., 2009; Cheng et al., 2018). There are 180 polyploidy events inferred in the angiosperm lineage, with many lineages having several WGD events yet returning to diploidy and fractionating to a typical number of genes (One Thousand Plant Transcriptomes Initiative, 2019). For instance, the aquatic carnivorous plant *Utricularia gibba*, which has the smallest published genome, has undergone three WGD events since its last common ancestor tomato (*Solanum lycopersicum*) yet maintains 25,000 genes due to fractionation (Ibarra-Laclette et al., 2013). In maize (*Zea mays*), which has experienced a recent tetraploidy and return to diploidy, the less dominant subgenome that has lower gene expression shows biased fractionation through intra-chromosomal recombination (Woodhouse et al., 2010; Schnable et al., 2011). However, in *Brassica rapa*, it was found that core clock genes are preferentially retained after the whole-genome triplication, suggesting an increase in circadian dosage may play an important role in crop domestication (Lou et al., 2012; McClung, 2021).

The ultimate consequence of WGD followed by fractionation is the extensive differences in gene order observed across plant genomes; generally, only closely related plant genomes retain genes in a similar order or syntenic blocks (Zhao and Schranz, 2019). However, even in syntenic blocks shared across species, gene order is thought to be randomly organized functionally across eukaryote chromosomes in contrast to prokaryotes where genes are often organized in functional arrays, or operons (Rocha, 2008). However, with an ever-increasing number of high-quality genomes and analytical tools it has become clear that there is in fact some level of gene clustering in eukaryotes and that some gene order is conserved evolutionarily (Supplemental Note S1; Hurst et al., 2004; Michalak, 2008; Winter et al., 2016; Foflonker and Blaby-Haas, 2021). The consequence of “gene neighborhoods” in eukaryotes is that either genes are co-expressed like bi-directional promoters in humans (Adachi and Lieber, 2002; Trinklein et al., 2004), or genetic linkages are preserved against allele shuffling by recombination like essential genes in yeast (Pál and Hurst, 2003). In plants, gene neighborhood have only been found for metabolic pathways (Osbourn, 2010; Kautsar et al., 2017; Nützmann et al., 2018, 2020; Bharadwaj et al., 2021), and co-expressed genes (Williams and Bowles, 2004; Zhan et al., 2006; Chen et al., 2010).

It was previously noted that *CCA1* and *PRR9* are in genetic linkage on Chromosome 2 (Chr02) in Arabidopsis (Michael et al., 2003; Lou et al., 2012), and later that *RVE4* and *PRR7* are linked on Chr05 (Michael et al., 2008b). However, despite the orthology of these *sMYB–PRR* gene pairs, these regions are not in syntenic blocks in Arabidopsis (Lou et al., 2012). Similar *sMYB–PRR* linkages have been noted in other eudicot species such as cranberry and blueberry, and monocot species such as duckweed (*Wolffia*, *Lemna*, *Spirodela*; Michael et al., 2020; Abramson et al., 2022; Kawash et al., 2021). These results suggest the *sMYB–PRR* linkage is evolutionarily conserved and that it may predate the land plants. Therefore, a syntenic ortholog analysis was conducted across the green lineage to elucidate the extent of evolutionary linkage between *sMYB–PRR* as well as other genes involved in the circadian clock.

## Results

### Syntenic paralogs of Arabidopsis circadian clock genes

For the purpose of exploring the relationship between *CCA1–PRR9* and *RVE4–PRR7*, a simplified model of their interactions was developed (Figure 1A; Shalit-Kaneh et al., 2018); more detailed models can be found in recent reviews (McClung, 2021; Steed et al., 2021). In general, *PRR7/9* are negatively and positively regulated by *CCA1/LHY* and *RVE4/8*, respectively, while in turn *PRR7/9* negatively regulate both. Consistent with these reciprocal functions, loss-of-function mutants *cca1* and *lhy* result in short FRP (<24 h), while loss-of-function *prr7*, *prr9*, *prr7/9*, and *rve4/6/8* result in long FRP (>24 h; Figure 1A; Supplemental Note S1). *CCA1*, *LHY*, *RVE4*, and *RVE8* have been shown to be expressed at dawn or shortly after. In contrast, *PRR9* has been shown to be expressed 4 h after dawn (Zeitgeber Time 4; ZT4) and *PRR7* at ZT7 (Figure 1, B and C; Supplemental Figure S1). Therefore, *PRR9* and *PRR7* peak several hours after their syntenic pairs of *CCA1* and *RVE4*, respectively.

The tight linkage of both sets of *CCA1–PRR9* and *RVE4–PRR7* paralogs suggested this could be mechanistically important to the daily timing of the clock. Additional linked genes and syntenic blocks were searched for using a collection of 61 circadian clock and light signaling genes (Supplemental Table S1; Lou et al., 2012). There were five gene pairs that were found within 12 genes of one another: *CCA1–PRR9* (4), *RVE4–PRR7* (3), *PIF–PHYA* (4), *PHYB–LKP2* (12), and *SRR1–BOA* (1) (gene pair and number of intervening genes; Supplemental Note S1). In addition, 38% (23) of genes were found in syntenic blocks with 14 in syntenic paralog pairs, and six genes in the syntenic pairs not on the circadian clock gene list (Supplemental Table S2). As has been reported before, the core clock genes *CCA1–LHY*, *RVE3–RVE5*, *ZTL–LKP2* are in syntenic blocks (Lou et al., 2012) as well as *RVE8*-*RVE4* (Figure 1, D and E; Supplemental Figure S2 and Supplemental Table S2; Shalit-Kaneh et al., 2018). However, no syntenic relationships were identified between the closely linked genes, nor specifically the *CCA1–PRR9* and *RVE4–PRR7* blocks. This result suggests that the *CCA1–PRR9* and *RVE4–PRR7* blocks were differentially inherited, possibly through distinct WGD and fractionation events.

Arabidopsis has experienced three polyploidy events termed lambda (***λ***), beta (***β***), and alpha (***α***) (Jiao et al., 2014). Based on Ks (synonymous substitutions) for the syntenic blocks, *CCA1–LHY* and *ZTL*-*LKP2* emerged from the ***λ*** whole-genome triplication (WGT) ∼150 million years ago (mya); *LUX–BOA*, *RVE3–RVE5*, and *CHE-TCP7* emerged from the ***β*** ∼75 mya; and *RVE4–RVE8* and *FLC–MAF5* emerged in the most recent ***α*** WGD ∼50 mya (Supplemental Table S3). This means that *CCA1–LHY* paralogous copies have been purged during the ***β*** and ***α*** events to maintain a single copy of each while *RVE4–RVE8* emerged recently, consistent with the two gene families representing two distinct evolutionary trajectories. A pairwise Ks analysis of both the *sMYB* and *PRR* families suggested the paralogs in each family are evolutionarily distant while *RVE4–RVE8* are relatively young since it is the result of the most recent ***α*** WGD event (Supplemental Tables S3 and S4). The synteny analysis and the Ks analysis both suggest that the *sMYB–PRR* linkage arose earlier than the Arabidopsis lineage.

### *sMYB–PRR* linkage arose in *Amborella*, the sister lineage to flowering plants

**Figure 2:**
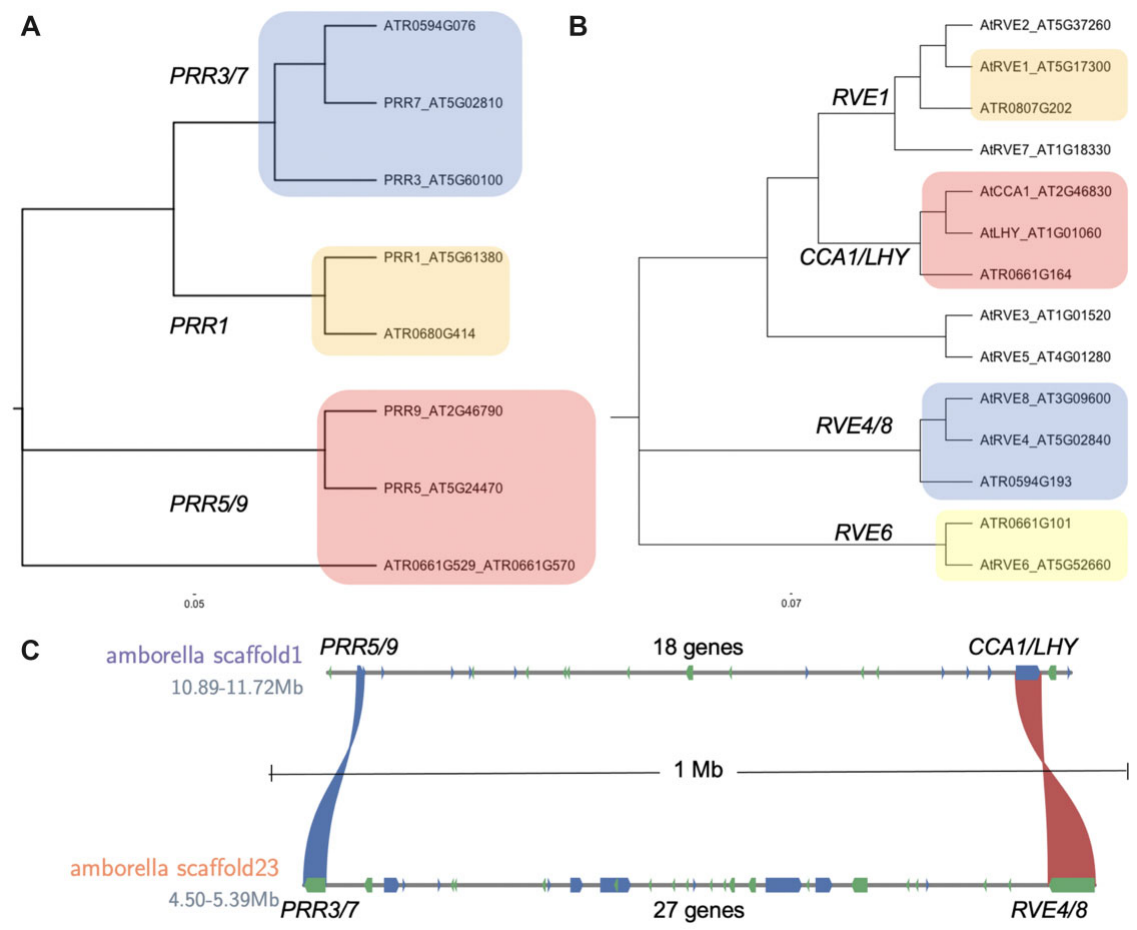
The *sMYB–PRR* linkages in *Amborella trichopoda*. The *Amborella* (*Amborella trichopoda*) and *Arabidopsis* (*A. thaliana*) A, *PRR* and B, *sMYB* phylogenetic trees resolve the specific relationships. C, *Amborella* scaffold1 and scaffold23 have the *CCA1/LHY–PRR5/9* and *RVE4/8–PRR3/7* linkages 18 and 27 genes apart, respectively. The *PRR* (blue ribbon) and *sMYB* (red ribbon) paralogs are the only syntenic genes in the region; the other genes (forward, blue; reverse, green) are depicted on the scaffolds.

*Amborella trichopoda* is the single living representative of the sister lineage to all other extant flowering plants and lacks a WGD event, which makes it attractive for tracing the lineage of gene families (Amborella Genome Project, 2013). *Amborella* only has three *PRR* proteins that are orthologous to Arabidopsis: *PRR1*, *PRR3/7*, and *PRR5/9*; the *PRR5/9* ortholog was split during gene prediction as two genes (ATR0661G529_ATR0661G570; Figure 2A). In addition, *Amborella* has four *sMYB* genes that are orthologs of Arabidopsis *CCA1/LHY*, *RVE4/8*, *RVE1/2/7*, and *RVE6* consistent with the fact that the ancestral plant has both *CCA1/LHY* and *RVE4/8* orthologs (Figure 2B; Sharma et al., 2017; Shalit-Kaneh et al., 2018). *Amborella* does not have a *RVE3/5* pair, which could mean that the pair arose from *RVE6* as suggested (Lou et al., 2012).

The fact that the *RVE4/8* ortholog existed before the monocot-eudicot split also suggested that the *sMYB–PRR* linkage may represent an ancestral state. Scanning the *Amborella* genome revealed that ATR0661G529_ATR0661G570 (*PRR5/9*) and ATR0661G164 (*LHY/CCA1*) were co-located 18 genes apart, separated by 800 kb on scaffold1 (Figure 2C). Likewise, ATR0594G076 (*PRR3/7*) and ATR0594G193 (*RVE4/8*) were co-located on scaffold23 separated by 27 genes and 900 kb (Figure 2C). Consistent with the organization in Arabidopsis, the linkages between the two *sMYB–PRR* clusters are present in *Amborella* suggesting that this linkage is an ancient arrangement of these core clock genes. Genetic linkages among *PIF3–PHYA*, *PHYB–LKP2*, and *SRR1–BOA* were also identified in Arabidopsis, yet only *PIF3–PHYA* was also found in *Amborella* (Supplemental Figure S3). Therefore, some of these genetic linkages may result from associations forming only in Arabidopsis, while the fact that the *CCA1–PRR9*, *RVE4–PRR7*, and *PIF3–PHYA* linkages date back to *Amborella* suggesting they could be evolutionarily important.

### *sMYB–PRR* and *PIF3–PHYA* linkages are inherited and closer in flowering plants

**Figure 3:**
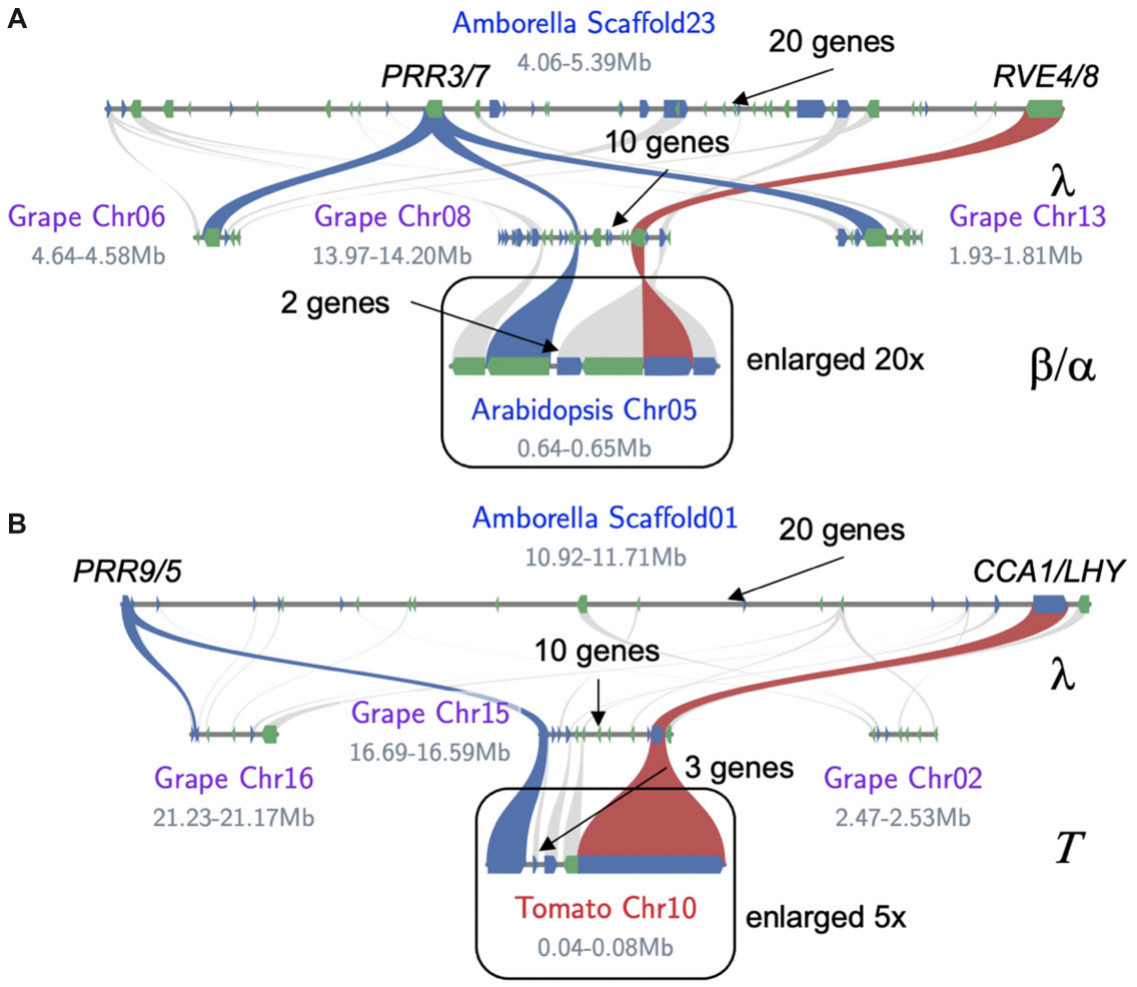
Syntenic *sMYB–PRR* pairs converge over evolutionary time. A, Syntenic blocks between *Amborella* (*Amborella trichopoda*), grape (*Vitis vinifera*), and Arabidopsis (*A. thaliana*). There are three syntenic blocks between *Amborella* and grape as a result of the λ WGT. All three copies of *PRR3/7* (blue) are retained on grape Chr06, Chr08, and Chr13, while only one *RVE4/8* (red) is retained on Chr08, which results in the *sMYB–PRR* linkage 10 genes apart. Grape Chr08 is syntenic to Arabidopsis Chr05 where *RVE4–PRR7* are 2 genes apart as a result of the ***β*** and ***α***; the Arabidopsis region is enlarged 20× to see the two intervening genes. B, Syntenic blocks between *Amborella* (*Amborella trichopoda*), grape (*Vitis vinifera*), and tomato (*Solanum lycopersicum*). Grape Chr08 is syntenic to tomato Chr10 where *RVE4–PRR7* are three genes apart as a result of the ***Τ*** (Tomato); the tomato region is enlarged 5× to see the three intervening genes. The other syntenic genes (forward, blue; reverse, green) are depicted in the homologous chromosomal regions (gray).

Grape (*Vitis vinifera*) has been used extensively to unravel evolutionary relationships in the eudicot lineage since it only contains the ***λ*** WGT event (Jiao et al., 2014). If the *sMYB–PRR* linkages were evolutionarily important then it might be expected that they were shared after distinct rounds of WGD. Syntenic orthologs between *Amborella* and grape were identified for both *CCA1/LHY–PRR5/9*, *RVE4/8–PRR3/7* and *PIF3–PHYA* pairs, with grape having 9, 10, and 12 intervening genes, respectively, fewer compared to the 18, 27, and 19, respectively, in *Amborella* (Figure 3A; Supplemental Figures S3 and S4). Whereas the *CCA1/LHY* and *RVE4/8* linkages are fractionated to one copy each, similar to Arabidopsis, *PRR5/9* and *PRR3/7* are retained in three and two copies respectively in grape (Supplemental Figure S4). These results suggest that there is a selective pressure to retain the *sMYB–PRR* linkage, possibly as a single copy each, and bring the linkage closer together.

Arabidopsis has experienced two additional WGD after the ***λ*** WGT event shared with grape, which provides an additional opportunity to test how the *sMYB–PRR* linkage is evolving. Grape and Arabidopsis share one synthetic block between *RVE4/8* and *PRR3/7* (Figure 3A). The *CCA1/LHY* and *PRR5/9 linkage* does not exist between grape and Arabidopsis because grape does not have a *CCA1* ortholog, and the *LHY–PRR* association has been lost (fractionated) in Arabidopsis (Supplemental Figure S5). The *RVE4/8–PRR3/7* linkage is yet again reduced from 10 genes in grape to 2 genes in Arabidopsis. Arabidopsis is in the rosid clade of the angiosperms, as is grape, which could mean that the *sMYB–PRR* linkage is specific to this clade. Tomato (*Solanum lycopersicum*), which is in the asterid clade of angiosperms and has an independent WGD event (***Τ***), was also found to retain the *sMYB–PRR* linkage with the distance between *RVE4/8* and *PRR3/7* reduced to three genes (Figure 3B). Thus, not only are the *sMYB–PRR* and *PIF3–PHYA* linkages inherited from the *Amborella* lineage in syntenic blocks, but the genetic linkages also moves progressively closer together suggesting that during fractionation these linkages are preferentially retained.

### *sMYB–PRR* and *PIF3–PHYA* linkages are conserved across angiosperms except the Poaceae

**Figure 4:**
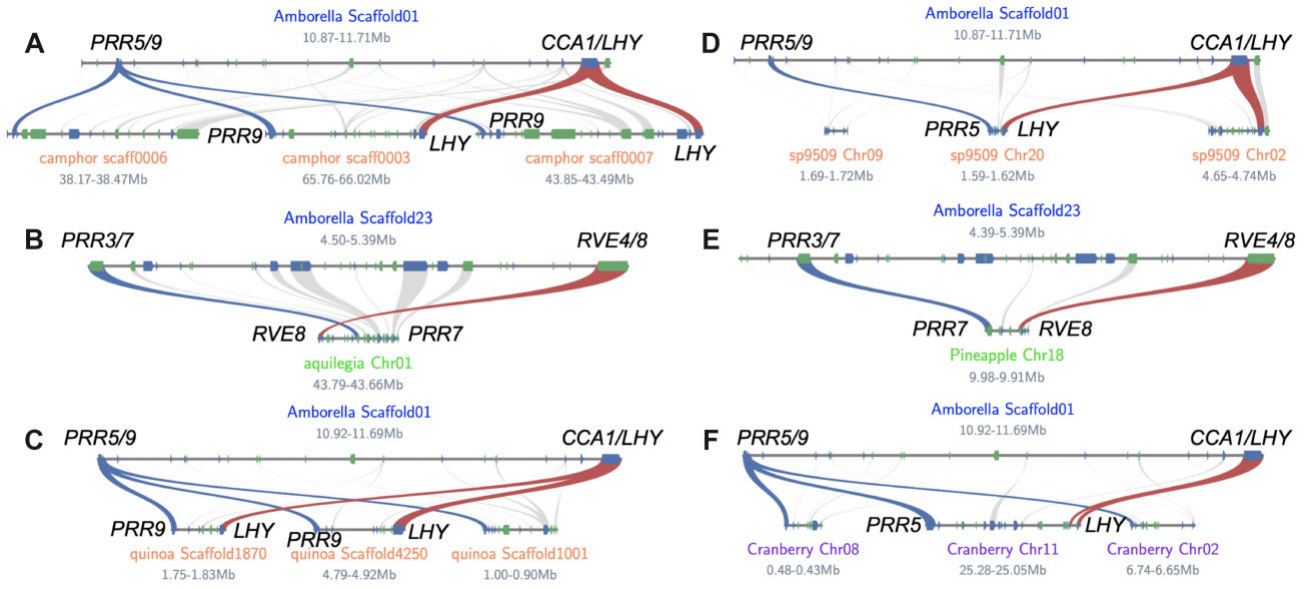
The *sMYB–PRR* linkage is conserved across Angiosperms. A, Two *LHY* (red) and *PRR5/9* (blue) linkages were retained in camphor (*C. camphora*; Magnoliids). B, One *RVE4/8* (red)*–PRR3/7* (blue) was retained in aquilegia (*A. coerulea*; Ranunculales). C, Two *LHY* (red) and *PRR5/9* (blue) linkages were retained in quinoa (*C. quinoa*; Caryophyllales). D, One *LHY* (red)*–PRR5/9* (blue) linkage was found in sp9509 (*S. polyrhiza* clone 5909; Araceae). E, One *RVE4/8* (red)*–PRR3/7* (blue) was found in pineapple (*Ananas comosus*; Commelinids). F, One *LHY* (red)*–PRR5/9* (blue) was found in cranberry (*Vaccinium macrocarpon*; Asterid); *LHY* is tandemly duplicated (TD) on Chr11. The other syntenic genes (forward, blue; reverse, green) are depicted in the homologous chromosomal regions (gray).

Several plant genome databases pre-compute syntenic block information, which provides a broader view of whether the *sMYB–PRR* and *PIF3–PHYA* linkages are generally retained. The PLAZA 4.5 dicot database was searched for *sMYB* and *PRR* orthologous genes and the presence of linkages (Supplemental Table S5; Van Bel et al., 2018). First, this analysis confirmed that only the Brassicacae have *CCA1*, while all other lineages lack *CCA1* and only contain *LHY* (Supplemental Figure S5). This result is consistent with the lack of *CCA1* in the grape lineage and the fact that it arose in the progenitor to Arabidopsis as a result of the ***λ*** WGT event. Of the eudicots, monocots, bryophytes, lycophytes, and algae in the PLAZA 4.5 dicot database, 62% had at least one *sMYB–PRR* linkage. This value (62%) is probably a conservative estimate due to the quality of genomes and the possibility that the *sMYB–PRR* linkage could be more than 10 genes in some species; for example, the *Amborella* linkages are not detected in this analysis because they are ∼20 genes apart. 50% of the species had both *sMYB–PRR* linkages, while slightly more than half (56%) had only *RVE4/8–PRR3/7* linkages and only 19% of those had more than one of either *sMYB–PRR* linkage (Supplemental Table S5). A similar analysis with the PLAZA 4.5 monocot database revealed that only *Spirodela*, pineapple, palm and orchid retained the *sMYB–PRR* linkage while revealing that all grasses (Poaceae) tested have lost the linkage sometime after the sigma (***σ***) WGD shared by pineapple and the grasses (Figure 4; Supplemental Figure S6; Ming et al., 2015; VanBuren et al., 2015).

A more comprehensive dataset was published recently that looked at the microsynteny of 123 plant species spanning angiosperms using a “syntenic ortholog network” approach (Zhao et al., 2021). Consistent with the results presented with other methods, *CCA1/LHY*, *RVE4/8*, *PRR3/7*, *PRR5/9*, *PIF3*, and *PHYA* were found in syntenic families. *PHYA* was alone in a syntenic family, while *PIF3* was part of a family that includes Arabidopsis *HFR1*, *PIL1*, and *PIL2* (Supplemental Tables S6 and S7). The *PRR3/7* and *PIF3* families contained 98.4% and 96.7% of the 123 plant species in syntenic blocks, respectively, which placed these syntenic families in the top 0.5% of all families for the number of species represented (Supplemental Table S7). In addition, both families shared blocks with *Amborella*, which was only found in 7.6% of families, making the *PRR3–PRR7* and *PIF3* families not only conserved but also found early in the angiosperm lineage. The other families (*CCA1/LHY*, *RVE4/RVE8*, *PRR5/PRR9*, and *PHYA*) were also in the 7.6% of families rooted in *Amborella* (share syntenic blocks) consistent with the fact that all of the families were also highly conserved over time. However, in contrast these other families only had syntenic blocks in between 69% and 81% of the 123 plant species. The absence of syntenic blocks in these families compared to *PRR3/7* and *PIF3* was primarily driven by the lack of these genes being found in syntenic blocks in the grasses (Poaceae; Supplemental Table S6). The *PHYA* family was also not found in syntenic blocks in the other monocots as well as the Solanaceae, Cucurbitaceae, and Malvaceae. Most species had more than one syntenic block for all of the families consistent with a history of WGD and retention of these blocks. The median for *CCA1/LHY*, *RVE4/8*, and *PHYA* was one syntenic block per species, while *PRR3/7*, *PRR5/9*, and *PIF3* was two syntenic blocks per species.

Although a high percentage of the species had these genes in syntenic blocks, the *LHY/CCA1–PRR5/9*, *RVE4/8–PRR3/7*, and *PIF3–PHYA* linkages could have been lost due to differential fractionation, yet 61%, 56%, and 57% retained at least one and 11%, 9%, and 12% retained more than one linkage, respectively (Supplemental Table S6). While many of the species with multiple linkages were polyploid (cotton, *Gossypium hirsutum*; *Camelina sativa*), soybean had the highest number of retained linkages across all three gene sets as found in other datasets. About 73% of species had at least one of the *LHY/CCA1–PRR5/9* and *RVE4/8–PRR3/7* linkages, and 53% had one of these linkages as well as the *PIF3–PHYA* linkage; 34% of species had all three linkages. The grasses (Poaceae) had completely lost all three linkages despite *PRR3/7* and *PIF3* being found in syntenic blocks. Considering that the grasses make up 13% of the species, the tight linkage between clock genes was found in a high number of nongrass species (Supplemental Table S6).

The “syntenic ortholog network” analysis revealed that many of the linkages across species were syntenic with *Amborella* despite distinct and numerous polyploidy events. In general, the syntenic blocks detected between *Amborella* and other species were very small (just containing the *sMYB–PRR* or *PIF3–PHYA* linkages), suggesting that there is selective pressure to retain these genes in syntenic blocks even as surrounding genes are fractionated. For the *CCA1LHY–PRR5/9* and *RVE4/8–PRR3/7* linkages there are eight different combinations possible (Supplemental Figure S7), all of which were detected across an array of evolutionarily distinct monocots and eudicots: *Cinnamomum camphora* (Ranunculales), *Aquilegia coerulea* (Magnoliids), *Manihot esculenta* (Malpighiales), *Cuscuta australis* (Convolvulaceae), *Chenopodium quinoa* (Caryophyllales), *Vaccinium macrocarpon* (Asterid), *Apostasia shenzhenica* (Asparagales), *Ananas comosus* (Poales), *Cocos nucifera* (Arecales), *Elaeis guineensis* (Arecales), and *Spirodela polyrhiza* (Araceae; Figure 4; Supplemental Figures S6 and S7; Zhang et al., 2017; Sun et al., 2018a; Ong et al., 2020; Kawash et al., 2021; Mansfeld et al., 2021; Yang et al., 2021b). In *Kalanchoe fedtschenkoi* (Saxifragales), a CAM photosynthesis plant that partitions carbon capture by TOD, the *LHY–PRR5* linkage has completely converged so the two genes are next to one another (no genes in between; Yang et al., 2017). These results are consistent with the *sMYB–PRR* linkage being a general feature of flowering plant genome evolution.

### Serial retention in soybean of the *sMYB–PRR* and *PIF3–PHYA* linkages

In the broad analysis of *sMYB–PRR* linkages in the PLAZA 4.5 database as well as the 123 angiosperm synteny network, soybean (*Glycine max*) had the most linkages with a total of six: four *CCA1/LHY–PRR5/9* and two *RVE4/8–PRR3/7* (Supplemental Table S5); it has also retained all four *PIF3–PHYA* linkages (Supplemental Figure S3). Soybean has experienced two WGDs 59 mya and a recent one 13 mya; after these WGD events, 50% of genes are still retained in syntenic pairs (Zhao et al., 2017). However, soybean has retained more than triple of the *sMYB–PRR* linkages compared to its close relatives chickpea (*Cicer arietinum*), barrel clover (*Medicago truncatula*), and mung bean (*Vigna radiata*), which have retained one of each for a total of two (Supplemental Table S5).

**Figure 5:**
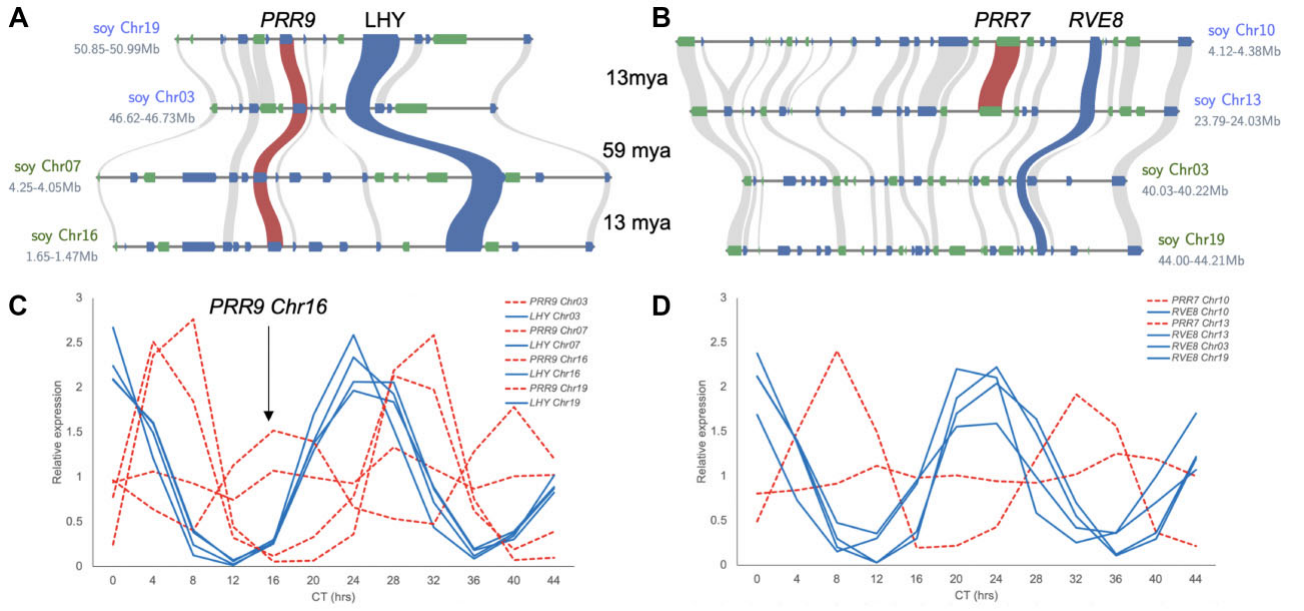
The *sMYB–PRR* linkages are preferentially retained in soybean. A, In Soybean (*Glycine max*; Williams82) the *LHY* (blue) and *PRR9* (red) linkage is retained over two WGDs 59 million years ago (mya) between Chr03–Chr07 and 13 mya between Chr03–Chr19 and Chr07–Chr16. B, In contrast, only two *RVE8* (blue) and *PRR7* (red) linkages are retained on Chr13–Chr10 after the most recent WGD 13 mya, while the other two copies are lost due to fractionation. The other syntenic genes (forward, blue; reverse, green) are depicted in the homologous chromosomal regions (gray). C, Expression of the soybean *PRR9* (red) and *LHY* (blue), and D, *PRR7* (red) and *RVE8* (blue) orthologs in continuous light and temperature over 44 h (Circadian Time; CT).

Like other species outside of the Brassicaceae, soybean does not have *CCA1* nor *RVE4*, yet has four copies of *LHY* and *RVE8*, in addition to two copies of *PRR7* and four of *PRR9*. Syntenic block analysis revealed that these genes formed the six different *sMYB–PRR* linkages (Figure 5). Leveraging Ks to date the syntenic blocks revealed that both linkages were retained after the WGD 59 mya, and that the *LHY–PRR9* linkage was retained after the WGD 13 mya while the *RVE8-PRR7* was fractionated. The similarity in fractionation between Chr03 and Chr19 suggested that the *PRR7* was fractionated before the WGD 13 mya that resulted in the retention of *RVE8* but the loss of two *sMYB–PRR* linkages. While soybean retains all four syntenic linkages of *PIF3–PHYA*, the blocks have diverged since the WGD 59 mya resulting in the linkages on Chr10 and Chr20 being seven and ten genes apart respectively and the linkages in Chr03 and Chr19 being four genes apart (Supplemental Figure S3). *PHYA* has been reported to be the gene underlying two soybean maturity group (MG) loci E3 and E4 (Nissan et al., 2021), which were found on Chr19 and Chr20 and separated by 56 mya.

Reanalysis of a recently published RNA-seq circadian time course in soybean revealed that while all of the *sMYB* (*LHY* and *RVE8*) in the linkages retained the morning-specific phase (Circadian Time 0; CT0) similar to Arabidopsis, the phases of the *PRRs* were variable (Figure 5, C and D). Specifically, the *PRR9* on Chr16 is expressed in the evening (CT17) compared to the morning expression of the other paralogs (CT6; Figure 5C). Only the *PIF3–PHYA* linkage on chr20, which is the MG E4 locus, cycles with a similar TOD expression as Arabidopsis with *PHYA* peaking at dusk (CT8) and *PIF3* peaking in the middle of the night (CT17; Supplemental Figure S8). The other *PIF3–PHYA* linkages, including the MG E3 locus on Chr19, have very low expression and are not predicted to cycle. In contrast to the *LHY/CCA1–PRR5/9* and *RVE4/8–PRR3/7* linkages that have morning to midday expressions, the *PIF3–PHYA* linkage peaks midday to midnight. Recently it was shown that knocking out all four of the *LHY* paralogs using CRISPR*–*CAS9 impacts plant architecture resulting in smaller plants and reduced internode length (Cheng et al., 2019). These results coupled to the preferential retention of the *sMYB–PRR* and *PIF3–PHYA* linkages over two rounds of WGD suggest this association could be of importance and a target for future soybean development via chronoculture (Steed et al., 2021).

### Maize and the loss of *sMYB–PRR* linkage

**Figure 6:**
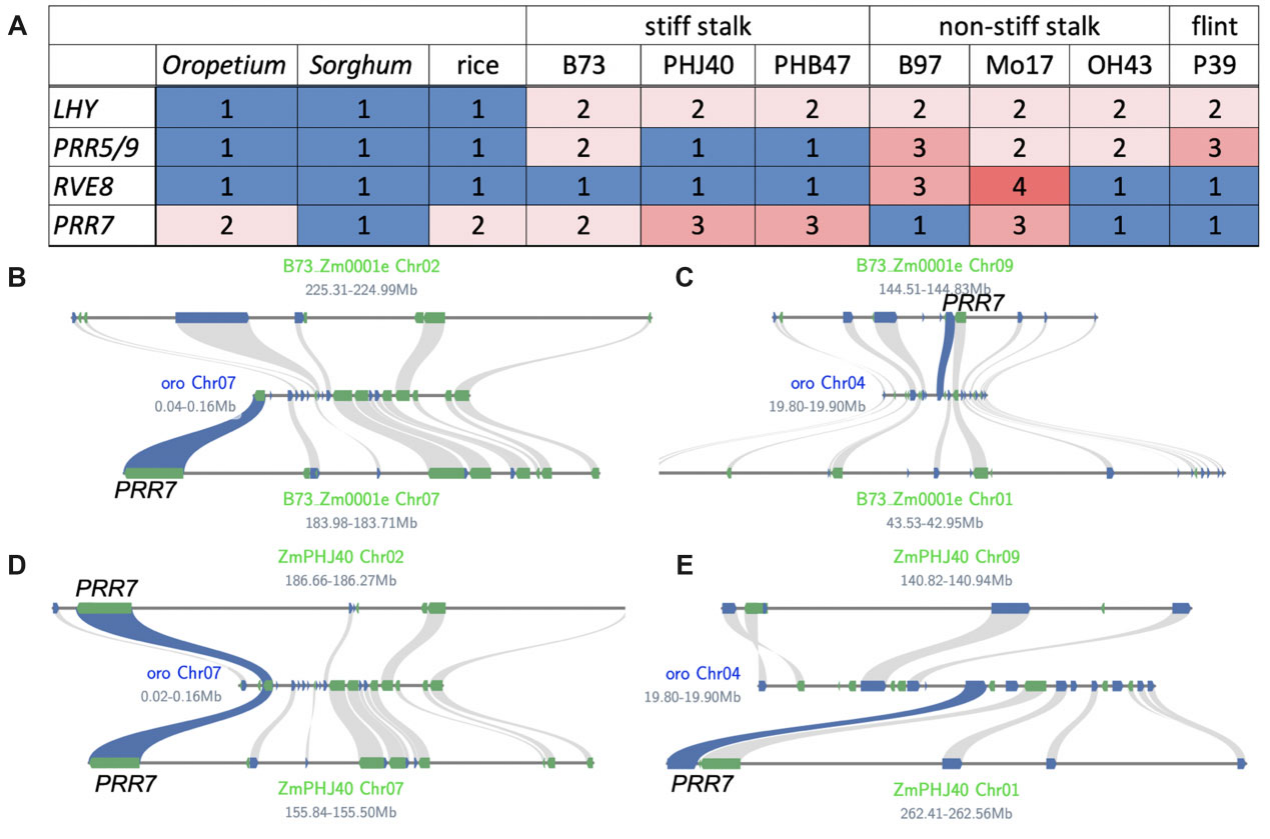
PAV of the *sMYB–PRR* genes in maize heterotic groups. A, Number of *LHY*, *PRR5/9*, *RVE8*, and *PRR7* copies across *Oropetium* (oro; *Oropetium thomaeum*, *Sorghum* (*S. bicolor*), rice (*O. sativa*), stiff stalk maize (B73, PHJ40 and PHB47), non-stiff stalk maize (B97, Mo17, OH43), and flint maize (P39). Squares are colored to draw contrast to the numbers: >1 (red) and = 1 (blue). B, C, The four maize (B73; Zm0001e) syntenic regions with the two *PRR7* (blue) copies found in *Oropetium* (oro) on Chr07 (B) and Chr04 (C). B73 only has two copies of *PRR7* on Chr07 and Chr09; the other two have been lost through fractionation. D and E, The maize line PHJ40 (ZmPHJ40) has three copies of *PRR7*; two copies are retained in syntenic blocks with oro on Chr07 and Chr02, the latter of which has been lost in B73 (B). One *PRR* copy is found in the oro Chr04 syntenic block on Chr01, which is the opposite found in B73 (C), consistent with all four copies of *PRR7* segregating in heterotic groups.

The loss of the *sMYB–PRR* linkage in the economically and agriculturally important grasses provided an opportunity to probe the importance of the genetic linkage. *Oropetium* (*Oropetium thomaeum*) had one copy each of *LHY*, *PRR5/9*, and *RVE8*, while it retained two copies of *PRR7*, all on different chromosomes (Figure 6; Supplemental Figure S6). The same was true for rice (*O.sativa*) and *Sorghum* (*Sorghum bicolor)*, except that *Sorghum* only had one *PRR7* copy (Figure 6A); similar results were found for *Setaria italica*, *Brachypodium dustachyon*, and other grasses for which high-quality genomes exist (Lai et al., 2020). However, maize (*Z.mays*; B73), which has experienced a recent WGD, retained two copies of each gene and has a slightly different pattern with only one *RVE8* (Figure 6A). A closer look compared to *Oropetium* revealed that not only was the second *RVE8* fractionated, but *PRR7* matched the two *Oropetium* chromosomal locations and the second copy of *PRR7* was fractionated (Figure 6, B and C). Since maize lines have a high level of presence absence variation (PAVs; Springer et al., 2009; Sun et al., 2018b), it is possible that the fractionation of these genes represented real differences in the content of *sMYB* and *PRR* between lines.

Maize lines have been developed into specific inbred heterotic groups such as stiff stalk (SS), nonstiff stalk (NSS), and flint (F) that are marked by high levels of PAVs, and when crossed, form the commercial hybrids that display “hybrid” vigor due to heterosis resulting in high yields and large plants (Bornowski et al., 2021). Looking at the *sMYB* and *PRR* genes across each of the heterotic groups in high-quality maize genomes revealed PAVs in these clock genes except *LHY*, which always had two copies resulting from the most recent WGD (Figure 6A; Hirsch et al., 2016; Sun et al., 2018b; Bornowski et al., 2021; Hufford et al., 2021). While the majority of maize lines retained two copies of *PRR7* syntenic to *Oropetium*, some had three as a result of a retention on Chr02; PHJ40 was unique in that it retains the copy on Chr01 instead, which suggested that all four *PRR7* copies resulting from the ancient WGD were differentially segregating across maize heterotic groups. In contrast, *RVE8* had one syntenic copy across all lines tested, but the additional copies in the NSS (MO17 and B97) were interspersed, suggesting they were duplicated in a manner other than WGD. When there were two copies of *PRR5/9*, they were syntenic pairs resulting from the most recent WGD, while only one copy represented a fractionation (PHJ40 and PHB47) and three copies (B97 and P39) were the result of a nonsyntenic dispersed duplication. These results suggested that the ability in maize to inherit different versions of the *sMYB/PRR* paralogs is important in the grasses and provides a clue as to why the *sMYB–PRR* linkage was broken.

### Origin of the *sMYB–PRR* and *PIF3–PHYA* linkages

**Figure 7:**
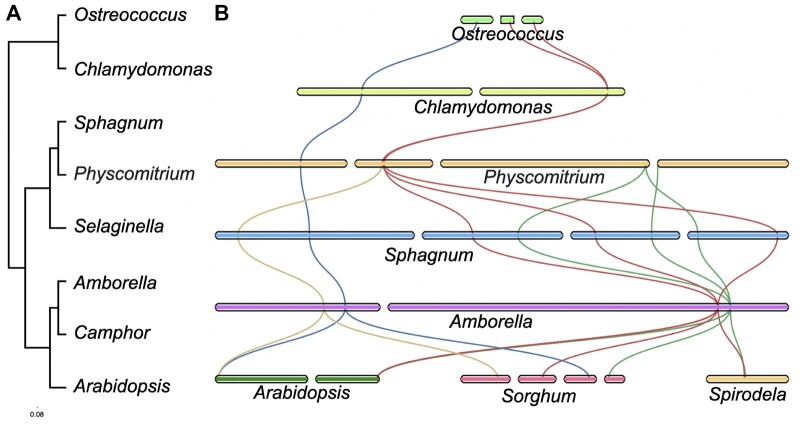
The *sMYB–PRR* linkage arose in the bryophyta. A, Phylogenetic tree spanning the green tree of life from green algae to flowering plants. Green algae: *Ostreococcus* (*O. lucimarinus*), and *Chlamydomonas* (*C. reinhardtii*); Bryophytes and Lycophytes: *Sphagnum* (*S. angustifolium*), *Physcomitrium* (*P. patens*), and *Selaginella* (*S. lepidophylla*); and flowering plants *Amborella* (*A. trichopoda*), *Camphor* (*C. camphora*), and Arabidopsis (*A. thaliana*). B, chromosomal view of the *RVE4/8* (blue), *CCA1/LHY* (green), *PRR5/9* (red), and *PRR3/7* (yellow) over evolutionary time. *Sorghum* (*S. bicolor*) and *Spirodela* (*S. polyrhiza*).

Recently it was shown that *RVE8* and *LHY* genes date as far back as unicellular green algae, and that these genes have antagonistic roles in the clock’s response to the environment (Shalit-Kaneh et al., 2018). The circadian clock in *Ostreococcus tauri*, a green unicellular picoalage, is controlled by a simple two component negative feedback loop of one *sMYB* and *PRR* (Corellou et al., 2009). In *O. tauri* and the closely related *O. lucimarinus*, there was one *sMYB* that clusters with *LHY* and two that clustered with *RVE* (Supplemental Figure S9). *Ostreococcustauri* had one *PRR* gene, while *O. lucimarinus* had two copies, which were the result of a WGD, all of which have been called *PRR1-like* (Corellou et al., 2009). However, based on a protein-based phylogenetic analysis, they were situated between the *PRR1* and *PRR5/9* clades (Supplemental Figure S9). All five genes were located on different chromosomes in both *O. lucimarinus* and *O. tauri*, indicating that even though both the *sMYB* and *PRR* clades were present, these two algae did not share the *sMYB–PRR* linkage (Figure 7). This was also true in the other chromosome resolved algae such as *Micromonas pusilla* (CCMP1545) and *Chlamydomonas reinhardtii*, both of which had two *sMYB* and one *PRR*, but they were found on separate chromosomes (Figure 7).

The charophyte (green algae), bryophyte (liverwort, hortwort, moss), and lycophytes (spike-moss) lineages all include both *sMYB* clades and *PRRs (*Linde et al., 2017; Li et al., 2018; Ferrari et al., 2019; Wickell et al., 2021). However, no *sMYB–PRR* linkage was detected in these genomes at either short or long distance; many of these genome assemblies were not chromosome-resolved, which could mean the linkage would be missed if it were on the scale found in *Amborella*. In addition, two chromosome-resolved moss genomes of *Physcomitrium patens* (formerly *Physcomatrella patens*) and *Ceratodon purpureus* had the *sMYB* and *PRR* on separate chromosomes and therefore clearly unlinked (Lang et al., 2018; Carey et al., 2021). A chromosome-resolved genome assembly of a bryophyte was available for *Sphagnum angustifolium* (formally *Sphagnum fallax*), which provided an opportunity to evaluate a third distinct moss genome (Meleshko et al., 2021). There was an expansion of both the *sMYB* and *PRR* gene families with seven and five respectively, four of which were located on the same chromosome (Figure 7). There were three *LHY/CCA1–PRR3/7* linkages on linkage group (LG)04, LG10, and LG15, that are 8, 8, and 12 Mb apart, respectively. The fourth was a *RVE6–PRR3/7* combination 12-Mb apart on LG02 (Figure 7). A similar situation was observed for *PIF3–PHYA*, where three pairs were found 8-, 7-, and 7-Mb apart on LG06, LG07, and LG12, respectively. While these are not closely linked like in Arabidopsis, the fact that they were on the same chromosome much like *Amborella* was suggestive that this may represent a linkage that predated the embryophyta (land plants).

Only recently have fern genomes (Li et al., 2018) and high-quality gymnosperm genomes (Scott et al., 2020) become available to assess the core circadian clock genes in the context of a genome. The *Azolla* and *Salvinia* genomes contained both *CCA1/LHY* and *RVE* clades with one and three, respectively, as well as *PRR1* and *PRR3/7*. However, none of these were in linkage on the contigs/scaffolds; once again this was possibly due to the fact that they are not assembled at a chromosome scale and they may be at the same or greater distance (∼12 Mb) as *Sphagnum* (*S*. *angustifolium*). A similar problem was encountered with gymnosperm genomes since they are between 5 and 30 Gb in size (Michael, 2014). However, it has been shown that the core circadian genes are conserved in conifers (Nose and Watanabe, 2014). Recently, a high-quality chromosome-resolved 8-Gb genome was published for the Giant Sequoia (*Sequoiadendron giganteum*; Scott et al., 2020). The Sequoia genome had a *RVE4/8–PRR3/7* linkage 12-Mb apart (51 genes) on Chr07 similar to that found in *Sphagnum* and collinear with *Amborella* (Supplemental Figure S7). Along with *Sphagnum*, the presence of *sMYB* and *PRR* on the same chromosome in the gymnosperm suggested that the linkage emerged before flowering plants.

**Figure 8:**
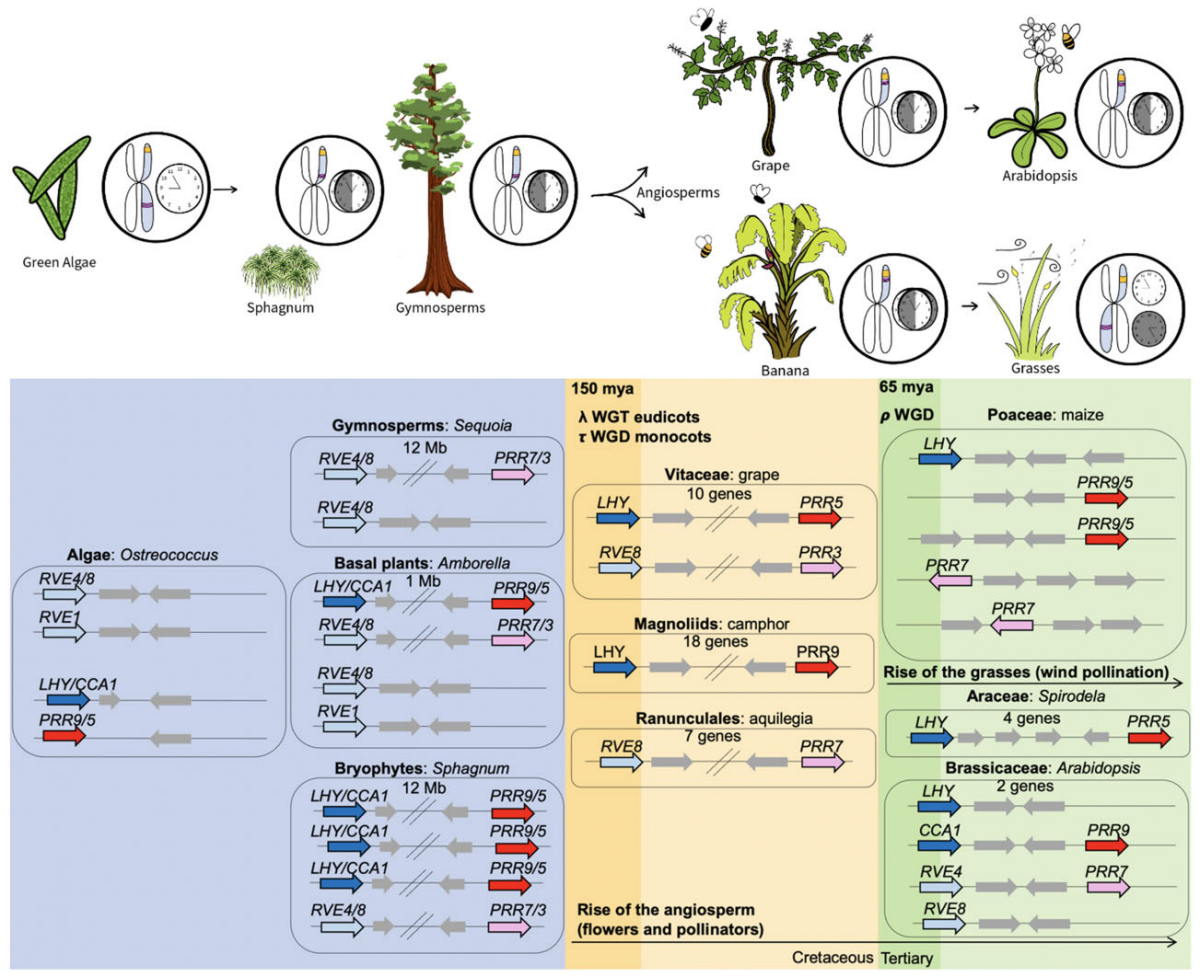
Model of the *sMYB–PRR* linkages across the green lineage. The green alga (*Ostreococcus lumimarinus*) has *RVE4/8* and *RVE1* (light blue), *LHY/CCA1* (blue), and *PRR9/5* (red), and these are all found on separate chromosomes; intervening genes are included (gray). Starting with *Sphagnum* (*Sphagnum angustifolium*) in the bryophyte lineage *sMYB* and *PRR* gene combinations are found on the same chromosomes 12-Mb apart. Similarly, the *RVE4/8–PRR3/7* linkage is found with Giant Sequoia (*Sequoiadendron giganteum*) also 12-Mb apart on the same chromosome. In *Amborella*, both *sMYB–PRR* linkages are found closer together at 1 Mb. In the early angiosperm lineages that emerged in the Cretaceous (orange box) of *Vitis vinifera* (grape; Vitaceae), *Cinnamomum camphora* (camphor; Magnoliids), and *Aquilegia coerulea* (aquilegia; Ranunculales) the genes are 7–18 genes apart. Lineages that emerged after the Cretaceous/Tertiary (K/Pg) boundary (green box) such as Arabidopsis (*A. thaliana*; Brassicaceae) and spirodela (*Spirodela* polyrhiza; Araceae) display genetic linkages that are two to four genes apart, while genes are found on separate chromosomes in the grasses like maize (*Zea mays*; Poaceae). The **λ** WGT in the eudicots, and the ***τ*** WGD in the monocots, as well as the ***ρ*** WGD shared in the grasses are indicated (Cheng et al., 2018).

Taken together these results showed that there was a progression from algae, where the *sMYB* and *PRRs* (and *PIF3–PHYA*) were on separate chromosomes, to angiosperm where they were found on the same chromosome separated by only several genes (Figure 8). The genetic linkages were found on the same chromosomes as early as the bryophytes (*Sphagnum*) and gymnosperm, and then progressively moved closer together from 12-Mb apart in *Amborella*, to 10–20 genes in early angiosperm lineages (grape, *C.camphora*, *A.coerulea*), and finally within 0–4 genes in plants that have more recent WGDs (Arabidopsis, *S.polyrhiza*). The exception in the angiosperm lineage were the grasses (Poaceae), where the *sMYB* and *PRR* (and *PIF3–PHYA*) were found on different chromosomes (Figure 8). The initial close genetic linkage between the genes coincides with the “rise of the angiosperm” in the Cretaceous 150 mya where angiosperms experienced an explosion of phenotypic as well as species diversification outcompeting the gymnosperms and ferns (Axelrod, 1952; Regal, 1977; Condamine et al., 2020; Ramírez-Barahona et al., 2020). This event also roughly coincided with the ***λ*** WGT in the eudicots, and the ***τ*** WGD in the monocots (Cheng et al., 2018). Furthermore, the progression of the genetic linkages moving even closer together in most species, and moving to other chromosomes in the grasses, coincided with the Cretaceous*–*Tertiary (K/Pg) boundary that was marked by several natural disasters and a large number of polyploidy events including the ***ρ*** (rho) WGD shared in the grasses (Figure 8; Fawcett et al., 2009; Li et al., 2018). It is possible that these events played a role in shaping the history of the genetic linkage of the circadian and light signaling genes.

## Discussion

The *LHY*/*CCA1–PRR5/9*, *RVE4/8–PRR3/7*, and *PIF3–PHYA* gene pairs reflect evolutionary conserved associations that emerged as early as the bryophyte lineage and progressively moved closer together in tighter genetic linkage in flowering plants. Most (>70%) plant lineages retained one copy of each *sMYB–PRR* or *PIF3–PHYA* linkages, and only 19% had more than one copy of any of the linkages (Supplemental Tables S5 and S6). While it has been found that co-expressed genes or genes in a shared metabolic pathway can be physically linked in plant genomes (Chen et al., 2010; Nützmann et al., 2018), to date evolutionarily conserved “gene neighborhoods” of differentially expressed nonorthologous genes in a developmental pathway have not been described in plants. In general, genes that are proximal in chromosome space (genetically linked) segregate together due to the low probability of a crossover event occurring between them; the closer genes are to one another, the more likely they will be inherited together. In plant genomes, it is more likely that genes will not share a similar order (synteny) over evolutionary time due to the active history of polyploidy, gene fractionation and reduction to a diploid state (Cheng et al., 2018; Qiao et al., 2019; Zhao and Schranz, 2019). The fact that the *sMYB–PRR* and *PIF3–PHYA* linkages were not only retained, but were brought into tighter linkage across all major clades of flowering plants (except the grasses), despite hundreds of distinct polyploid events, argues these associations are under selection to ensure some aspect of circadian clock function.

It is formally possible that these gene pairs are physically linked to ensure their co-regulation/co-expression. However, in Arabidopsis the phase of expression of *CCA1–PRR9*, *RVE4–PRR7*, and *PIF3–PHYA* under light and temperature cycles (LDHC) are 4, 8, and 12 h apart, respectively, and are differentially phased by distinct environmental conditions (Michael et al., 2008b). For instance, the phase of expression for *CCA1* and *PRR7* is constant regardless of entrainment, whereas *RVE4* and *PRR9* are shifted 4 h later under thermocycles alone (LLHC) (Michael et al., 2008b). *PIF3* and *PHYA*, are expressed antiphasic whenever thermocycles are present (LLHC or LDHC versus LDHH, short day or long day). One other expression-based explanation for the linkages could be a shared chromatin state to ensure they are expressed in the same tissue, cell type or developmental time; it has been shown that the evening complex generates a repressive chromatin state to control the expression of *PRR7* and *PRR9* (Tong et al., 2020). However, the linked gene pairs have distinct expression patterns over an array of conditions, cell types, developmental times and tissues (Waese et al., 2017), suggesting it is unlikely that a similar chromatin state fully explains their genetic linkage.

It is important to place the expression patterns of the *sMYB–PRR* and *PIF3–PHYA* linkages in context of other plant species. Both the *CCA1–PRR9* and *RVE4–PRR7* are specific to the brassicaceae since *CCA1* and *RVE4* are the result of specific WDG events in this lineage. In other species all combinations of the *sMYB–PRR* associations are present: *LHY–PRR9* (soybean), *LHY–PRR5* (wolffia, poplar), *RVE8–PRR7* (soybean, palm), and *RVE8–PRR3* (grape, cuscuta; Figure 4; Supplemental Figure S7). The nongrass monocot wolffia (*Wolffia australiana*) has one *LHY–PRR5* association with *WaPRR5* having a similar phase of expression to Arabidopsis *PRR9* peaking 8 h after lights on (Michael et al., 2020). In poplar (*Populus trichocarpa*), there are two *LHY–PRR5* genetic linkages; both genetic linkages cycle with similar phases as the Arabidopsis *CCA1–PRR9* with dawn and several hours after dawn peaks, respectively (Filichkin et al., 2011). However, much like *PRR9* in Arabidopsis, *PtPRR5* is sensitive to thermocycles and its phase overlaps with *PtLHY* when a thermocycle is present (Filichkin et al., 2011). These results highlight that not only has the genetic linkage been conserved across an array of different WGD events, but that the timing of expression may also provide a clue as to the importance of this tight genetic linkage.

The *PRRs* were originally characterized by their “waves of expression” starting with *PRR9* peak expression shortly after dawn, followed by *PRR7*, *PRR5*, *PRR3*, and *PRR1* separated by 2–3 h, and it was speculated that *PRR9* and *CCA1* expression may be coordinated by *PIF3* via the G-box (CACGTG; Matsushika et al., 2000). An updated model demonstrated that the waves of *PRR* expression during the day are required to restrict the growth promoting activity of the *PIFs* to the evening (Martín et al., 2018). The *PIFs* and *PRRs* physically interact and compete for the G-box during the day ensuring photoperiod-specific growth (Soy et al., 2016; Martín et al., 2018; Li et al., 2020). Since the *PRR5/7/9* directly bind the promoter of *CCA1* and *PRR7* binds the promoter of *RVE4* via the G-box (Liu et al., 2016; Martín et al., 2018), the timing of the *PRRs* and *PIFs* together actively ensure the environment specific expression of the *sMYB* in linkage. Moreover, *CCA1* directly binds the promoters of *PRR1/5/7/9* via the Evening Element (AATATCT; Nagel et al., 2015; Kamioka et al., 2016), which suggests the specific *sMYB–PRR* linkages form stable feedback loops ensuring balanced timing. However, *PIF3* is not part of the core circadian clock since its loss-of-function mutant does not result in any circadian defects (Stephenson et al., 2009), and it is genetically separable from the core circadian clock since loss of *PRR7* also does not alter *PIF3* expression (Soy et al., 2012; Martín et al., 2018). *PIF3* is regulated by the photoreceptors *PHYA* and *PHYB*, which both control stability and inhibit its binding activity (Soy et al., 2012; Yoo et al., 2021). Therefore, the *PIF3–PHYA* “growth” linkage ensures the genetic consistency of how input light information is translated into output of growth, while the *sMYB–PRR* linkages ensure a balanced timing.

The “environmental robustness” model is proposed to explain the importance of the evolutionarily conserved genetic linkages: the *sMYB–PRR* and *PIF3–PHYA* linkages ensure the inheritance of a balanced and robust circadian clock for environment-specific growth. This model builds on an updated circadian clock model where the two different clades of *sMYBs* play opposing roles in controlling not only period length but also growth through the *PIFs* (Figure 1; Shalit-Kaneh et al., 2018). The *lhy/cca1* double mutant has very short FRP and reduced plant stature, while the *rve4/6/8 triple* mutant has a very long FRP and increased plant stature; the growth phenotypes are dependent on *PIF4* and *PIF5* (Gray et al., 2017). However, the loss of both *lhy/cca1* and *rve4/6/8* (*lhy/cca1/rve4/6/8* quintuple) restores circadian period and plant stature to wild-type, yet with decreased robustness to different environmental conditions (Shalit-Kaneh et al., 2018). These results demonstrate that the different feedback loops of the circadian clock are “dispensable”, but are important for the plant to accurately anticipate daily changes in the environment to ensure growth tuned to their current location and time in the season. In addition, these results show that the role of the clock in controlling growth is dependent on the *PIFs*. The only study to indirectly test the importance of the *sMYB–PRR* genetic linkages, found that the loss of *CCA1*/*LHY* in the *PRR7* and *PRR9* background (*prr7/prr9/cca1-amiR/lhy-amiR*), results in a short circadian period much like the *cca1/lhy* alone (Salomé et al., 2010), consistent with *CCA1*/*LHY* being epistatic to and mediating *PRR7* and *PRR9* input. Taken together, along with the fact that *sMYB–PRR* pairs as well as *PRR–PIF* pairs form regulatory loops, the inheritance of specific *sMYB–PRR* and *PIF3–PHYA* would ensure the inheritance of a specific “circadian state”.

While the plant circadian clock acts to restrict biological activities to the correct time over the day for the specific location and season (Michael et al., 2003; Dodd et al., 2005), complete loss of the circadian clock, or arhythmic plants can grow just fine under ideal lab conditions (Schaffer et al., 1998; Wang and Tobin, 1998; Mizoguchi et al., 2002). For instance, the arhythmic *LHY* gain of function (overexpressor) is indistinguishable from wild-type under lab conditions (continuous light and temperature), yet has extremely small stature and early flowering plant under short-day conditions (Michael et al., 2008a), consistent with the circadian clock playing an important role to integrate external timing cues. Therefore, the clock is dispensable (under ideal conditions), but plays a fundamental role to integrate environmental signals to either restrict (“put on the brakes''), or promote (“press the gas”) biological processes to ensure that the plant is synchronized to the daily and seasonal changes in its local environment. This is very important because plants cannot move, and usually germinate close to the parental genotype that is optimized for that location. The genetics of the parental genotype are passed to the offspring providing them the best advantage for that local environment. Genetically linked circadian clock and light signaling genes such as *sMYB–PRR* and *PIF3–PHYA* may ensure that the proper timing parameters, or circadian state, are passed with fidelity to the offspring, regardless of the mating system (inbreeding versus outcrossing).

It is well established that the circadian clock provides enhanced fitness by enabling sessile plants to synchronize and thus anticipate the daily changes in their environment (Green et al., 2002; Dodd et al., 2005). For instance, it was found that the FRP of leaf movement in Arabidopsis accessions was correlated to the latitude of origin (Michael et al., 2003). Accessions from higher latitudes have longer periods, while accessions from lower latitudes have shorter or variable periods. One explanation for this is that Arabidopsis germinates late spring at higher latitudes and has a narrow window as the days get longer to reach maturity and flower before it gets too hot. In contrast, accessions at lower latitudes experience less of a contrast of temperature and increasing day lengths, and therefore, having a period closer to the external environment is advantageous. A similar relationship between latitude and FRP has also been noted in wild annual populations of mimulus (*Mimulus guttatus*), as well as domesticated soybean and tomato (Müller et al., 2016; Greenham et al., 2017). However, perennial mimulus populations displayed less variation in period length and FRP was not correlated with latitude, which provides support for the idea that the correlation between period and latitude allows annuals to accurately proceed through development during a changing season (Greenham et al., 2017). Tomato is a perennial, but mostly grown as an annual crop for production, which could also explain why domesticated varieties have an increase in FRP that would allow them to optimize growth during a typical growing season starting in the spring (Müller et al., 2016).

In environmental robustness framework, when two populations adapted to different environments mate, the offspring would inherit a mixed circadian state, and the hypothesis is that in the first filial (F_1_) generation the circadian clock would not gate growth and instead would “release the breaks”, resulting in rapid growth and flowering in the new environment regardless of resources. Generally, this is regarded as heterosis and has been shown to rely on the circadian clock (Ni et al., 2009; Shen et al., 2015; Ko et al., 2016; Yang et al., 2021a). Then in the F_2_ generation, plants with the optimal circadian state for the new environment have an advantage and generate more offspring. However, key to this scenario is how the circadian components are inherited. If core components of the clock are genetically linked then they will most likely be inherited together, with only very rare crossover events unlinking them. This will ensure that selfing or mating with close relatives will maintain a specific circadian state, while wide-crosses or outcrossing will result in potentially mixed but balanced circadian state that could provide advantage to the new offspring (F_1_) in a new environment.

One prediction of the environmental robustness model is that species that have a higher dependence on photoperiodic timing should have more linkages and/or they will be closer together. For instance, in the model obligate CAM plant *Kalanchoe*, which fixes up to 99% of its carbon during the night (Yang et al., 2017), the *sMYB–PRR* linkage has been fractionated and retained with zero intervening genes. In another example, the economically important crop soybean is a species that is highly photoperiod sensitive with varieties arranged in MGs based on their flowering time, which also correlate with circadian period length (Greenham et al., 2017). Soybean has at least twelve loci underlying MGs, of which two are core circadian clock genes (*EARLY FLOWERING 3*; *ELF3a* and *GIGANTEA*; *GIa*), three are flowering time genes (*FLOWERING LOCUS T*; *FT3a*; *FT1a* and *TERMINAL FLOWER1*; *TFL1b*) and two are the *PHYA* genes (*PHYA2*, *PHYA3*; Li and Lam, 2020; Nissan et al., 2021). The two *PIF3–PHYA* linkages have been identified as MG loci E3 and E4 (Li and Lam, 2020), and recent genome-wide association studies (GWAS) suggest other circadian linkages could be involved in flowering time (Kim et al., 2020). These results suggest that the E4 MG might be an evening specific association between *PHYA* and *PIF3*, and not just *PHYA*. However, it is becoming clear that the *PRRs* not in the genetic linkage also play an important role in agronomic traits; recently it has been shown through quantitative trait loci and GWAS that soybean *PRR3* is a target of domestication (Li et al., 2019b; Lu et al., 2020), which was also identified as a maturity locus involved in Sorghum flowering time (*ma1/SbPRR3*; Murphy et al., 2011). Soybean is a powerful example because it directly connects the genetic linkages and the circadian state (MG) that optimizes its ability to synchronize with its environment (Greenham et al., 2017).

Since the grasses have lost all of the genetic linkages, they provided an important counterpoint and refinement to the environmental robustness model. Monocots retain the genetic linkage up to pineapple in the poales, and then it is lost completely in the grasses (poaceae; Supplementary Table S6), and close relatives in the graminid clade. In contrast to soybean that retained most of its genetic linkages across both of its recent WGD, maize not only lost the linkages but displayed variable subgenome fractionation for *PRR5/9*, *RVE8*, and *PRR7* across different heterotic groups (Figure 6). *PRR7* was of particular interest because it is *HEADING DATE 2* (*HD2*) in rice and variation at this locus provides photoperiod and temperature sensitivity (Koo et al., 2013). All four *PRR7* copies resulting from the past two WGD events (∼75 mya and ∼12 mya) segregated across maize heterotic groups, with lines having different numbers from one to three (Figure 6). It has been shown that the genes in the less dominant and expressed subgenome are preferentially fractionated (Schnable et al., 2011), but it seems that the fractionation of *PRR7* across the heterotic groups displayed a distinct pattern. One explanation for the interesting *PRR7* segregation, leveraging the environmental robustness model, was that instead of generating a location specific circadian state, the diversity and chromosomal distribution of alleles generated plants with unrestricted growth to quickly grow and colonize a new location. In other words, when the circadian and light signaling genes were inherited together they restricted or “gated” growth for a specific local environment, whereas when the linkages were broken and differentially inherited growth was no longer gated and the plants displayed accelerated growth similar to hybrid vigor (heterosis). Supporting this interpretation, it has been shown that the circadian clock plays a role in hybrid vigor (Ni et al., 2009; Shen et al., 2015; Ko et al., 2016; Yang et al., 2021a), and it could be that this specific type of inheritance of the circadian clock linkages provides some clues as to the success of the grasses (Linder et al., 2018).

The fact that the circadian and light signaling genetic linkages either moved closer together or separated at specific times over plant evolutionary history provided an opportunity to speculate on their historical importance (Figure 8; Supplementary Figure S10). While there are hints of genetic linkages in the Sphagnum and gymnosperms, the tight linkage emerged in the angiosperm lineage. The emergence and then rise to dominance, in terms of morphological adaptation and number of species, of the angiosperm over the gymnosperms and ferns in the Cretaceous has long captivated biologists with Darwin referring to it as the “abominable mystery” (Soltis et al., 2009). One distinctive feature of the angiosperms is the flower, and the subsequent intimate relationship with specific pollinators, which enables angiosperms to maintain small local environmental-specific populations while also enabling the colonization of widely spaced microhabitats (Regal, 1977; Atamian et al., 2016). In the framework of the environmental robustness model, the tight linkage would ensure that the small local populations would be synchronized with their microhabitat as well as their pollinator. At the same time, the ability of animal pollinators to generate wide crosses between plants and transport seed to widely distributed “safe sites”, stimulated novel circadian and light signaling genes combinations, similar to the grasses, which led to evolutionary momentum and speciation. Therefore, the genetic linkage and pollinator interaction fostered speciation cycles: first, microhabitat synchronization with a specific circadian state; then wide dispersal of new genetic combinations and hybrid vigor to colonize a new location: followed by selection of a new circadian state and microhabitat specialization.

The next important event in the angiosperm lineage occurred at the Cretaceous/Tertiary boundary (K/Pg), which was marked by several natural disasters, a large number of polyploidy events and emergence of the grasses from other monocots. The grasses are arguably the most successful of the angiosperm by sheer number of species, diverse environments they inhabit (20% of the Earth’s terrestrial surface), and importance to caloric initiate of the world (Stanley, 1999; Edwards and Smith, 2010; Linder et al., 2018). While most angiosperms brought the circadian and light signaling genetic linkages even closer together after new rounds of WGD at the K/pg, the grasses took the opposite approach segregating the genes to different chromosomes (Figure 8; Supplementary Figure S10). The grasses emerged in the shady understory of forests and later colonized the open plains and diverse environments (Edwards and Smith, 2010). Some of the underlying traits that enabled the grasses to colonize, persist and transform their environment were shade tolerance, short generation times, starch-rich seed, long-distance wind pollination and dispersal, and resilience to disturbance (grazing and fire; Linder et al., 2018). It is possible that growth in the understory reduced the strict reliance on synchronizing with daily light/dark cycles, which is supported by recent results showing that grasses are more sensitive to thermocycles for TOD expression and growth (Matos et al., 2014; MacKinnon et al., 2019), and favored genetic combinations resulting in rapid growth (hybrid vigor) associated with unlinked circadian and light signaling genes. Moreover, grasses have adopted wind pollination and the unlinked genes would enhance genetic combinations and growth vigor as they invade and colonize new environments per the environmental robustness model (Friedman and Barrett, 2009).

## Conclusion

The circadian and light signaling genes were brought into tight linkage over evolutionary time and distinct plant lineages. These linkages represent “gene neighborhoods” of differentially expressed nonorthologous genes controlling a fundamental developmental pathway, which has not been described in plants previously. Considering the conservation of the circadian clock across the green lineage, the extent that biological processes are controlled in a TOD fashion and the enhanced fitness the circadian clock confers, the linkages most likely represent an important event in the evolution and biology of plants. The environmental robustness model, in light of different reproductive strategies, provides an explanation for both the tight genetic linkage as well as the loss of the linkages found in the grasses; the linkages can be inherited to confer a specific circadian state or be mixed, either through wide-hybrids, or as in grasses on separate chromosomes, to create circadian clock novelty, hybrid vigor, and speciation. Since the circadian clock plays a central role in domestication (McClung, 2021), and the grass and dicot crops have distinct inheritance of circadian clock genes, it will be important to consider the genetic linkage when developing novel varieties.

## Materials and methods

### Circadian clock genes

A list of core circadian clock genes, as well as light signaling and flowering time genes associated with the clock, from Arabidopsis (*A.thaliana*) were used to seed the initial analysis (Supplemental Table S1; Lou et al., 2012).

### Genomes

Genomes described were downloaded from Phytozome13 (https://phytozome-next.jgi.doe.gov/) and PLAZA (https://bioinformatics.psb.ugent.be/plaza/). When genomes were downloaded from other sources the primary reference was cited at the first mention of the species in the text.

### Gene family analysis

Proteins for species specifically mentioned in the text were evaluated for orthology using orthofinder (v2.3.11) using default settings (Emms and Kelly, 2015). Different combinations of species were run during multiple rounds of orthofinder to detect when the sMYB and PRR family relationships merged and split. In general, smaller clustering of closely related species resulted in the PRRs splitting into separate families (*PRR1* and the other PRRs), as well as *CCA1* (when it was present in the Brassicaceae)/*LHY* and the RVEs. Circadian families were manually compared to the results in the PLAZA databases: PLAZA dicot 4.5 and monocot 4.5 databases (Van Bel et al., 2018).

### Syntenic ortholog analysis

All syntenic analyses were conducted with either MCscan using the python version (https://github.com/tanghaibao/jcvi/wiki/MCscan-(Python-version) or CoGe SynMap (https://genomevolution.org/;Grover et al., 2017). All syntenic ortholog searches were conducted by all-by-all protein alignments using last with default settings (Kiełbasa et al., 2011). Resulting protein pairs were filtered using default settings of a C-score = 0.7, which optimized for protein pairs that are greater than 70% best match in either genome as described (Hasing et al., 2020). Both MCscan (Tang et al., 2008) and DAGchaniner (CoGe; Haas et al., 2004) were run requiring at least five syntenic pairs per 20 gene syntenic block, which is the default setting for CoGe SynMap (Grover et al., 2017). Similar syntenic block settings (five syntenic pairs per 25 gene syntenic block) were empirically validated across an array of angiosperm genomes (Zhao and Schranz, 2019). Syntenic plots were generated using the MCscan python version with default settings. Broad syntenic relationships across plant genomes were analyzed using the PLAZA dicot 4.5 and monocot 4.5 databases (Van Bel et al., 2018), as well as syntenic relationships identified across 123 high-quality plant genomes using a network approach based on MCscanX (Supplemental Note S1; Zhao et al., 2021). The results presented were consistent across all four platforms (MCscan python version, MCscanX, CoGe SynMap, and PLAZA).

### Expression analysis

Published Arabidopsis (Yang et al., 2020) and soy (Li et al., 2019a) RNA-seq datasets were downloaded from the Sequencing Read Archive and reanalyzed using the updated DIURNAL pipeline (https://gitlab.com/NolanHartwick/super_cycling) using the same reference genomes described in this study (Michael et al., 2020; Wickell et al., 2021). The resulting cycling matrix was used to plot cycling expression profiles.

### Accession numbers

Identification numbers for the circadian and light signaling genes discussed in this article can be found in Supplemental Table S1 (Lou et al., 2012).

## Supplemental data

The following materials are available in the online version of this article.

**Supplemental Note S1.** Circadian and light signaling evolutionary linkage

**Supplemental Figure S1.** Expression of core circadian clock genes in *Arabidopsis*.

**Supplemental Figure S2.** Syntenic orthologs and expression of core circadian clock genes.

**Supplemental Figure S3.** *PIF3* and *PHYA* linkage conserved back to *Amborella*.

**Supplemental Figure S4.** Syntenic *sMYB–PRR* pairs between *Amborella* and grape.

**Supplemental Figure S5.** *CCA1/LHY* lineage across monocots and eudicots.

**Supplemental Figure S6.** The *sMYB–PRR* syntenic block in pineapple reveal relationships across monocots.

**Supplemental Figure S7.** Different *sMYB–PRR* combinations are found across plant genomes with distinct WGD events.

**Supplemental Figure S8.** *PIF3–PHYA* are expressed at distinct times of day in *Arabidopsis* and soybean.

**Supplemental Figure S9.** Circadian clock and light signaling genes are duplicated in Ostreococcus.

**Supplemental Table S1.** Arabidopsis circadian clock, light signaling, and flowering time genes.

**Supplemental Table S2.** Syntenic regions for core circadian clock genes in *Arabidopsis*.

**Supplemental Table S3.** Synonymous substitution (Ks) across *Arabidopsis sMYB* proteins.

**Supplemental Table S4.** Synonymous substitution (Ks) across *Arabidopsis PRR* proteins.

**Supplemental Table S5.** Genetic linkages between *CCA1/LHY–PRR5/9* and *RVE4/8–PRR3/7* from PLAZA dicot 4.5.

**Supplemental Table S6.** Number of syntenic blocks and genetic linkages between *CCA1/LHY–PRR5/9*, *RVE4/8–PRR3/7*, and *PIF3–PHYA*.

**Supplemental Table S7.** Summary of syntenic blocks across 123 plant genome assemblies for circadian genes.

## Supplementary Material

kiac276_Supplementary_Data

## Notes

T.P.M. conceived the study, conducted analysis, and wrote the article.

The author responsible for distribution of materials integral to the findings presented in this article in accordance with the policy described in the Instructions for Authors (https://academic.oup.com/plphys/pages/general-instructions) is: Todd P. Michael (tmichael@salk.edu).
