# Supplementary material for: Core circadian clock and light signaling genes brought into genetic linkage across the green lineage
Source: Plant Physiol. 2022 Jun 8;190(2):1037–56. doi: 10.1093/plphys/kiac276 (PMC9516744; doi:10.1093/plphys/kiac276)
Supplement: kiac276_Supplementary_Data [file kiac276_supplementary_data.pdf]

Supplementary Note: Circadian and light signaling evolutionary linkage

Core circadian clock and light signaling genes brought into genetic linkage across the green lineage

Todd P. Michael[1]

[1]The Plant Molecular and Cellular Biology Laboratory, The Salk Institute for Biological Studies, La Jolla, CA 92037, USA

Corresponding author: Todd P. Michael; tmichael@salk.edu

Phenotypes of core circadian clock genes in *Arabidopsis*

The first plant circadian clock mutant described in *Arabidopsis* was *timing of cab expression 1* (*toc1*) that has short free-running period (FRP) under continuous conditions and early flowering phenotypes (Millar et al., 1995; Somers et al., 1998). It was later cloned and shown to encode *PSEUDO-RESPONSE REGULATOR* (*PRR1*) with conserved CCT (CONSTANS, CO-like, and TOC1) and REC (receiver) domains (Strayer et al., 2000). *TOC1/PRR1* is part of a five gene family including *PRR3*, *PRR5*, *PRR7*, and *PRR9* that is regulated in “circadian waves of expression” with peak gene expression at Zeitgeber (ZT, German for “time giver”) 13, 11, 8, 7 and 4 hours (hrs) after lights on (dawn) respectively in long day conditions (16 hrs light/8 hrs dark) (Matsushika et al., 2000; Michael et al., 2008). The loss of evening expressed *PRRs* (*PRR1/3/5*) result in short FRP, while the morning expressed *PRRs* (*PRR7/9*) result in a long FRP (Michael et al., 2003; Salomé and McClung, 2005). Loss of *PRR9* results in differential impact depending on the output rhythm assayed; there is a change in the phase of circadian regulated leaf movement, and core clock gene expression displayed a long FRP (Michael et al., 2003; Salomé and McClung, 2005). Similarly, *PRR7* is essential for the cycling of *CCA1* in the shoots but not in the roots, suggesting that it has tissue specific roles in circadian clock regulation (Nimmo and Laird, 2021). The double mutant combination of *PRR7* and *PRR9* (*prr7/9*) results in loss of temperature compensation and very long FRP, suggesting that they have independent functions in the circadian clock consistent with not being direct paralogs of one another or having similar expression (Figure 1; Supplemental Table S4) (Farré et al., 2005; Salomé and McClung, 2005).

The second family of core circadian clock genes identified were *LATE ELONGATED HYPOCOTYL* (*LHY*) and *CIRCADIAN CLOCK ASSOCIATED 1* (*CCA1*), which are part of a sub-family of the large myeloblastosis (MYB) transcription factors (TF) family defined by the “SHAQKYF” protein motif (*sMYB*) (Schaffer et al., 1998; Wang and Tobin, 1998). Both *LHY* and *CCA1* have peak expression at dawn regardless of entraining conditions (different photocycles and thermocycles) or under driven (diurnal) or free-run (circadian) conditions (Michael et al., 2008). Gain of function (overexpression) of either *LHY* or *CCA1* results in arrhythmic leaf movement, hypocotyl elongation, and gene expression of clock-controlled genes (Schaffer et al., 1998; Wang and Tobin, 1998). In contrast, loss of function of either result in a short FRP (3 hrs) and early flowering under short days (SD), although hypocotyl length and flowering time are wild type under long days (LD) (Green and Tobin, 1999; Alabadí et al., 2002; Mizoguchi et al.,

2002; Oda et al., 2007). The double mutant (*cca1/lhy*) results in an even shorter FRP (6 hrs) than the single mutant, early flowering and plants with very small stature, suggesting that these orthologs are partly redundant in the core clock (Mizoguchi et al., 2002; Lu et al., 2009; Salomé et al., 2010).

In addition to *CCA1* and *LHY*, the sMYB sub-family consists of eight other genes that share both the "SHAQKYF" motif as well as dawn-specific expression and thus were named *REVEILLE* (*RVE*) (Chaudhury et al., 1999). *RVE3*, *RVE4*, *RVE5*, *RVE6*, and *RVE8* form a sub-clade of the *RVEs* since they also share the LHY-CCA1-like (LCL) domain and hence have also been referred to as *LCL3*, *LCL1*, LCL4, *LCL2*, and *LCL5* respectively (Farinas and Mas, 2011). The *LCL* sub-clade are generally expressed at dawn like the other *RVEs* except *RVE6/LCL2* only cycles under LD and SD conditions (not under circadian free-run conditions), and *RVE4* peaks in the afternoon under thermocycles (Michael et al., 2008). In contrast, *RVE1*, *RVE2* and *RVE7* peak at ZT0 (dawn), ZT18 (midnight) and ZT07 (afternoon) respectively (Supplemental Figure S1) (Michael et al., 2008).

The first *RVE* gene described was an gain of function (overexpression) line of *RVE7*/*EARLY- PHYTOCHROME-RESPONSIVE 1* (*EPR1*) that didn't result in circadian period defects but did cause late flowering under LD and repressed its own expression consistent with it forming a slave oscillator (Kuno et al., 2003). Next, an overexpressing line of *RVE2/CIRCADIAN 1* (*CIR1*) was described that resulted in short FRP, delayed flowering, longer hypocotyls and reduced seed germination in the dark (Zhang et al., 2007). Similar to *RVE7*, loss of *RVE1* did not result in a defect in FRP, but it does result in changes in growth due to alterations in the auxin pathway (Rawat et al., 2009). The *LCL* sub-clade was the last to be described beginning with *RVE8/LCL5*; overexpression results in a shorter FRP and late flowering under both LD and SD, while loss of function causes a long FRP and early flowering (Farinas and Mas, 2011; Rawat et al., 2011). Loss of function of either of the closely related *RVE4/LCL1* or *RVE6/LCL2* does not result in a FRP change, but the double (*rve4/8*, 27 hrs; *rve6/8*, 26 hrs) or triple (*rve4/6/8*, 28 hrs) with *RVE8/LCL5* results in a progressively longer FRP (Figure 1) (Hsu et al., 2013). Loss of function of either *RVE3/LCL3* or *RVE5/LCL4* also do not result in FRP changes, while the double mutant (*rve3/5*) has a slightly shorter FRP and the quintuple mutant (*rve3/4/5/6/8*) has a even longer FRP (28 hrs) (Gray et al., 2017).

Since both the *sMYB* and *PRR* gene families are redundant, combinatorial mutation analysis provides clues as to significance of the *CCA1-PRR9* and *RVE4-PRR7* genetic linkages. One study has looked at the loss of the *CCA1-PRR9* linkage but in the context of other redundant genes *LHY* and *PRR7* (Salomé et al., 2010). The double mutant *prr7/9* results in a very long FRP, yet when *CCA1* or *LHY* are reduced using artificial microRNAs (amiR) silencing technology, the period is shortened almost to wild type FRP, while loss of both *CCA1* and *LHY* has a similar short FRP as the *lhy/cca1* (Salomé et al., 2010). These results suggest that the *CCA1-PRR9* linkage results in reciprocal impacts on FRP, but that *CCA1* and *LHY* are epistatic to *PRR7* and *PRR9* impacts on FRP.

In contrast to the *lhy/cca1* double mutant that results in plants with a smaller stature, the *rve4/6/8 triple* mutant results in larger plants and the increased growth that is dependent on *PIF4* and *PIF5* (Gray et al., 2017). However, loss of both *lhy/cca1* and *rve4/6/8* (*lhy/cca1/rve4/6/8* quintuple) restores the growth defect and FRP, suggesting that the the two clades of *sMYB* have reciprocal and dispensable roles in maintaining timing information and

growth (Shalit-Kaneh et al., 2018). So what are these specific feedback loops used for? While the *lhy/cca1/rve4/6/8* quintuple has restored growth and FRP, the circadian clock is less robust with decreased amplitude and suboptimal response to adverse environmental conditions (Shalit-Kaneh et al., 2018). Since *CCA1-LHY*-mediated temperature compensation requires both *PRR7* and *PRR9* (Salomé et al., 2010), it is possible that the *CCA1-PRR9* and *RVE4-PRR7* linkages represented inherited positive and negative respectively regulators of growth through the *PIFs* in a thermo and photo-sensitive way. To this end, the *PRR9* and *RVE4* phase of expression is shifted by 4 hours under thermocycles (while *CCA1* and *PRR7* are not), suggesting differential integration of thermocycle information (Michael et al., 2008).

Gene neighborhoods in plant genomes

Gene order in eukaryotes is generally poorly conserved resulting in seemingly random organization across chromosomes in contrast to prokaryotes where genes are often organized in functional arrays, or operons (Rocha, 2008). However, with more high-quality genomes and analytical tools it has become clear that there is in fact some level of gene clustering in eukaryotes and that some gene order is conserved evolutionarily (Hurst et al., 2004; Michalak, 2008). Two different studies across a collection of eukaryotic genomes spanning from plants to humans revealed that functionally and transcriptionally related genes are found in non-random clusters in the genome (Lee and Sonnhammer, 2003; Dávila López et al., 2010). In humans, bi-directional promoters play a role in proximally co-expressed genes (Adachi and Lieber, 2002; Trinklein et al., 2004). In yeast, essential genes are more likely to be found in clusters where the recombination rate is lower and this is independent of co-expression, suggesting that at some level genetics plays a role at preserving gene order (Pál and Hurst, 2003).

While many studies focus on identifying clusters based on functional or co-expression information, several tools have been developed to take an unbiased approach to find “gene neighborhoods,” or Proxiomal Ortholog Gene (POG) pairs of non-homologous genes (Winter et al., 2016; Marcet-Houben and Gabaldón, 2020; Foflonker and Blaby-Haas, 2021). Leveraging an evolutionary approach, up to 32% of the gene space across 341 fungal genomes are found in gene neighborhoods with many representing metabolic clusters (Marcet-Houben and Gabaldón, 2019). In algal genomes far fewer gene neighborhoods were identified, but they revealed several non-metabolic novel pathways (Foflonker and Blaby-Haas, 2021).

In plants a systematic look for gene neighbors has primarily been restricted to metabolic pathways (Osbourn, 2010; Kautsar et al., 2017; Nützmann et al., 2018; Nützmann et al., 2020; Bharadwaj et al., 2021), or co-expressed genes (Williams and Bowles, 2004; Zhan et al., 2006; Chen et al., 2010). Plants are special amongst the eukaryotes since they undergo extensive whole genome duplication (WGD) and polyploidy events followed by rounds of fractionation that greatly increases the random order of genes and decreases gene synteny across lineages (Vision, 2005; Cheng et al., 2018). For instance, it has been shown across an array of high-quality genomes of mammals and plants that only closely related plants retain a similar level of synteny that is found across all mammals (Zhao and Schranz, 2019). Therefore, the *sMYB-PRR* and *PIF3-PHYA* evolutionarily conserved non-homologous gene clusters involved in a genetic network (as opposed to a metabolic pathway) are the first to be described across plant genomes.

The evolutionary significance of the environmental robustness model

The increasing closeness of the gene linkages appears around the same time as the rise to dominance of angiosperms over gymnosperms and ferns during the Cretaceous (Condamine et al., 2020). The phenotypic and species diversity of the angiosperm has been attributed to the multiple rounds of whole genome duplication (polyploidy) and fractionation (Soltis et al., 2009), which is the process by which the gene linkages are moving closer together over evolutionary time. There are two forces at work here: first, polyploidy often brings together distant genomes (allopolyploidy), which is thought to be maintained due to hybrid vigor enabling the ability to thrive in harsh/disparate environments or an asexual lifestyle (Fawcett et al., 2009; Cheng et al., 2018). Second, polyploids are ultimately reduced back to diploids (Zhao et al., 2017), which must thrive in their specific environment, yet sex is risky because it would be easy to make an unwanted genetic combination for a local environment (Freeling, 2017).

It is thought that the major innovation that led to the dominance of angiosperm was the flower and the specific relationship that it fostered with pollinators (Supplementary Figure S10) (Regal, 1977). Therefore, the linkage of light and circadian genes ensures that plants are tuned to exploit their specific environments, inheriting the correct combination for their local conditions so they will grow optimally under different seasons (Michael et al., 2003; Dodd et al., 2005). Another burst of polyploidy occurred at the Cretaceous/Tertiary (K/Pg) boundary that coincided with several natural disasters (Fawcett et al., 2009), and at this time the light and circadian gene linkages moved closer together in almost all species test, except the grasses. This suggests that most plants “doubled down” on ensuring that the circadian system was inherited for a specific location; maybe the global decreasing temperature and carbon dioxide made it more likely that plants specially tuned for their environment would thrive and reproduce (Condamine et al., 2020).

At the same time, grasses became the most successful angiosperms and started to fill new ecological niches such as shaded forests and later open plains (Linder et al., 2018). Grasses are completely wind pollinated and flower at specific times of day (TOD) (Friedman and Barrett, 2009), suggesting they have taken the exact opposite route from other angiosperms and aggressively ensure every progeny has a new combination of circadian and light alleles. In essence, every pollination event represents a wide-hybrid that experiences heterosis or hybrid vigor, which enables it to outcompete populations in its new location. This strategy has been termed the “Viking syndrome” describing the ability of the grasses to colonize, persist and transform their environments (Linder et al., 2018). Taken together, the close genetic linkage favors animal pollination where specific circadian timing states are maintained; whereas the broken linkages favors wind pollination where diverse circadian states enable possible colonization of new environments.

Supplemental Figures

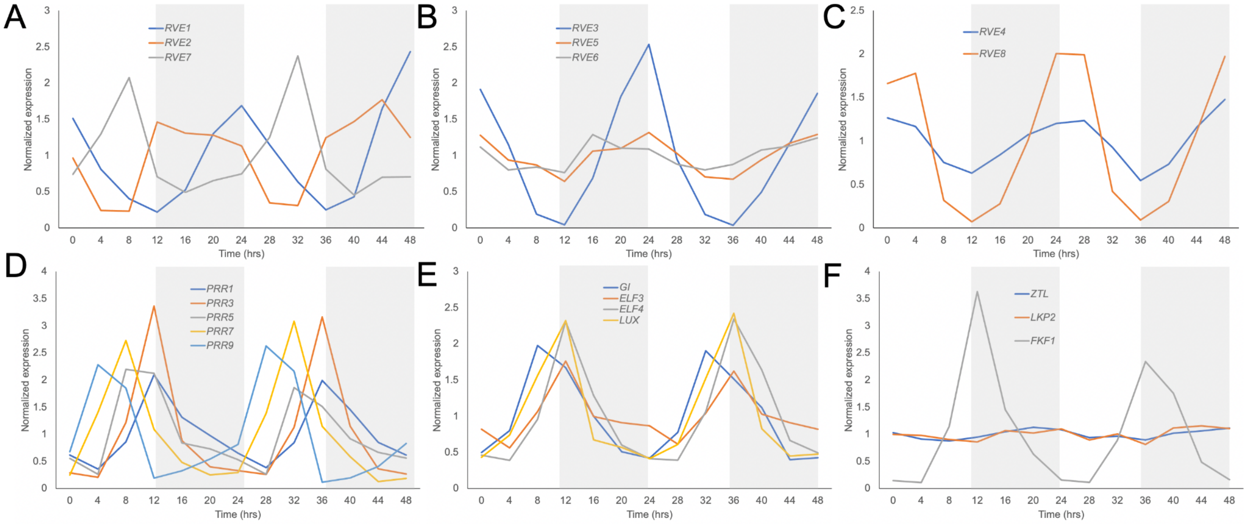

Supplemental Figure S1. Expression of core circadian clock genes in *Arabidopsis*. Core circadian clock genes grouped by gene family or function. A) *RVE1*(blue), *RVE2* (orange) and *RVE7* (grey). B) *RVE3* (blue), *RVE5* (orange), and *RVE7* (grey). C) *RVE4* (blue) and *RVE8* (orange). D) *PRR1* (blue), *PRR3* (orange), *PRR5* (grey), *PRR7* (yellow) and *PRR9* (aqua); E) *GI* (blue), *ELF3* (orange), *ELF4* (grey), and *LUX* (yellow). F) *ZTL* (blue), *LKP2* (orange) and *FKF1* (grey). Normalized RNA-seq expression was plotted over the day with grey boxes representing the dark period.

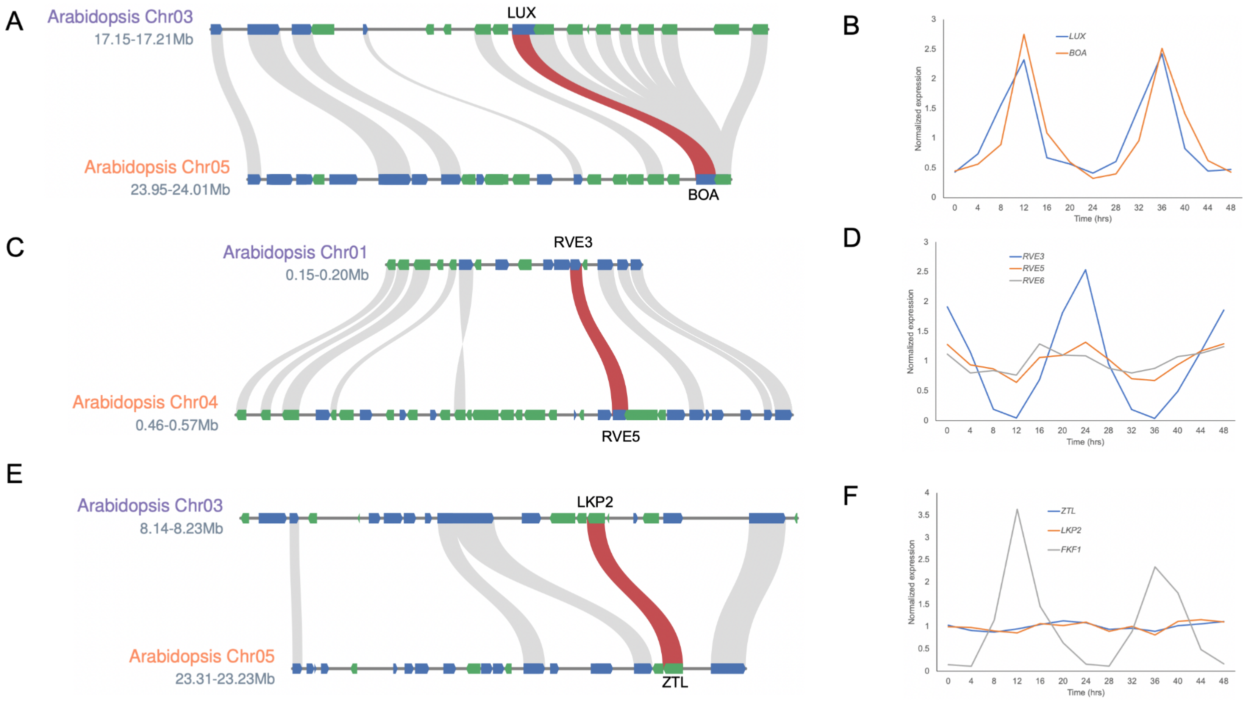

Supplemental Figure S2. Syntenic orthologs and expression of core circadian clock genes. A) The syntenic block for *LUX* (top) and *BOA* (bottom). B) The expression for *LUX* (blue) and *BOA* (orange). C) The syntenic block for *RVE3* (top) and *RVE5* (bottom). D) The expression *RVE3* (blue), *RVE5* (orange) and *RVE6* (grey). E) The syntenic block for *LKP2* (top) and *ZTL* (bottom). F) The expression of *ZTL* (grey), *LKP2* (orange) and *FKF1* (blue). Key syntenic relationships (red) and other syntenic genes (grey) for the entire syntenic block. In the syntenic plots the genes on the positive strand (blue) and negative strand (green).

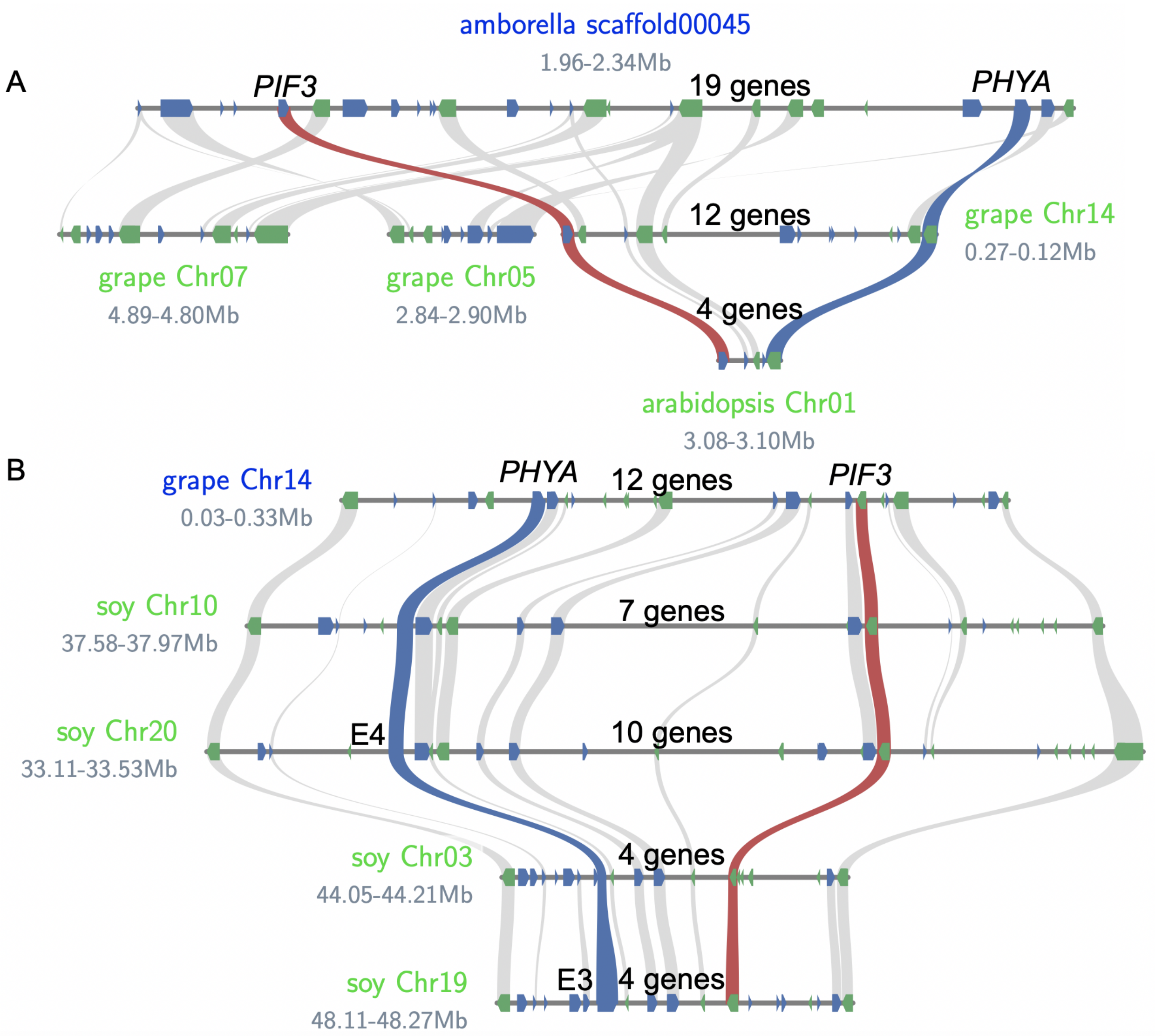

Supplemental Figure S3. *PIF3* and *PHYA* linkage conserved back to *Amborella*. *PIF3* syntenic relationships (red), *PHYA* syntenic relationships (blue) and other syntenic genes (grey) for the entire syntenic block. Genes on the positive strand (blue) and negative strand (green). A) Syntenic blocks between *Amborella* (*Amborella trichopoda*), grape (*Vitis vinifera*) and *Arabidopsis* (*Arabidopsis thaliana*). B) Syntenic blocks between grape (*Vitis vinifera*) and soy (Glycine max).

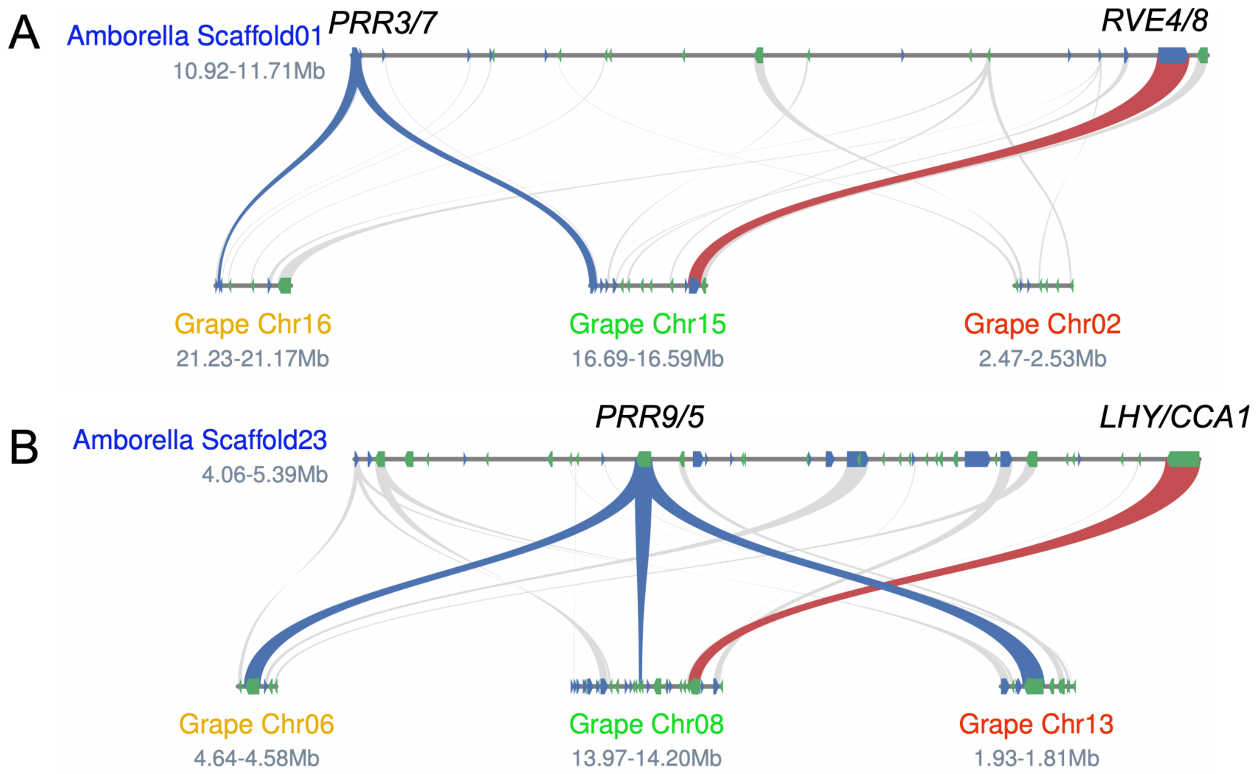

Supplemental Figure S4. Syntenic *sMYB-PRR* pairs between *Amborella* and grape. *LHY/CCA1* syntenic relationships (red), *PRR3/5/7/9* syntenic relationships (blue) and other syntenic genes (grey) for the entire syntenic block. Genes on the positive strand (blue) and negative strand (green). A) Syntenic blocks between *Amborella* (*Amborella trichopoda*) and grape (*Vitis vinifera*) for the *RVE4/8-PRR3/7*. B) A) Syntenic blocks between *Amborella* (*Amborella trichopoda*) and grape (*Vitis vinifera*) for the *LHY/CCA1-PRR5/9*.

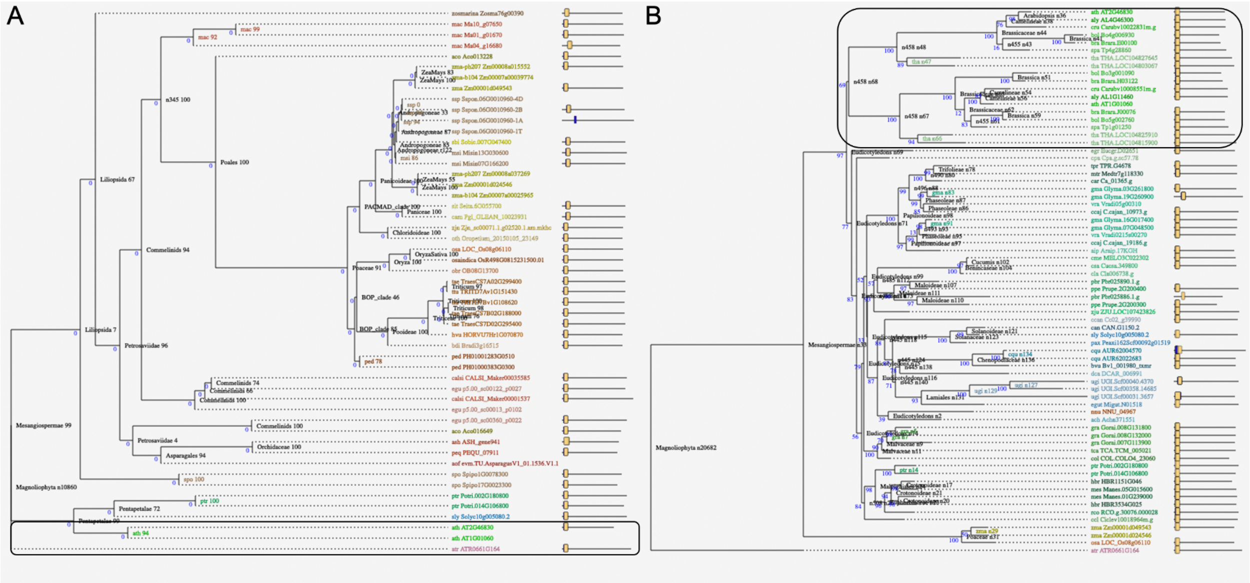

Supplemental Figure S5. *CCA1/LHY* lineage across monocots and eudicots. Both trees are pre-generated from the PLAZA dicot and monocot web pages. Gene names are on the tips of the tree and domain structure is depicted to the left. A) Monocot *LHY/CCA1* tree; and B) Eudicot *LHY/CCA1* tree. *CCA1* genes boxed.

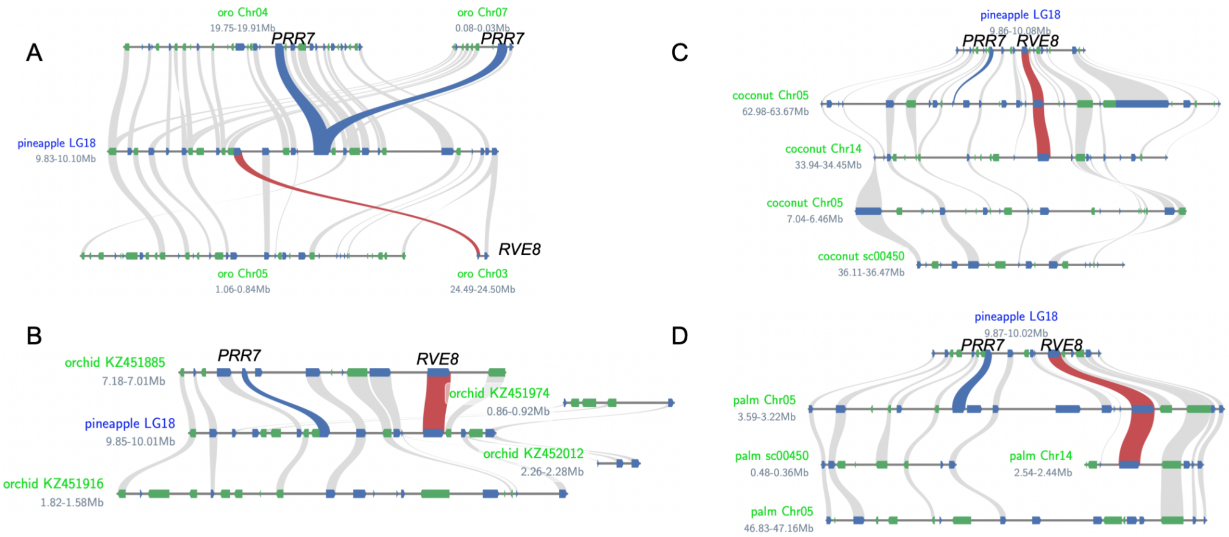

Supplemental Figure S6. The *sMYB-PRR* syntenic block in pineapple reveals relationships across monocots. *LHY/CCA1/RVE* syntenic relationships (red), *PRR3/5/7/9* syntenic relationships (blue) and other syntenic genes (grey) for the entire syntenic block. Genes on the positive strand (blue) and negative strand (green). A) Pineapple (*Ananas comosus*) versus oro (*Oropetium thomaeum*); B) pineapple versus orchid (*Apostasia shenzhenica*); C) pineapple versus coconut (*Cocos nucifera*); D) pineapple versus palm (*Elaeis guineensis*).

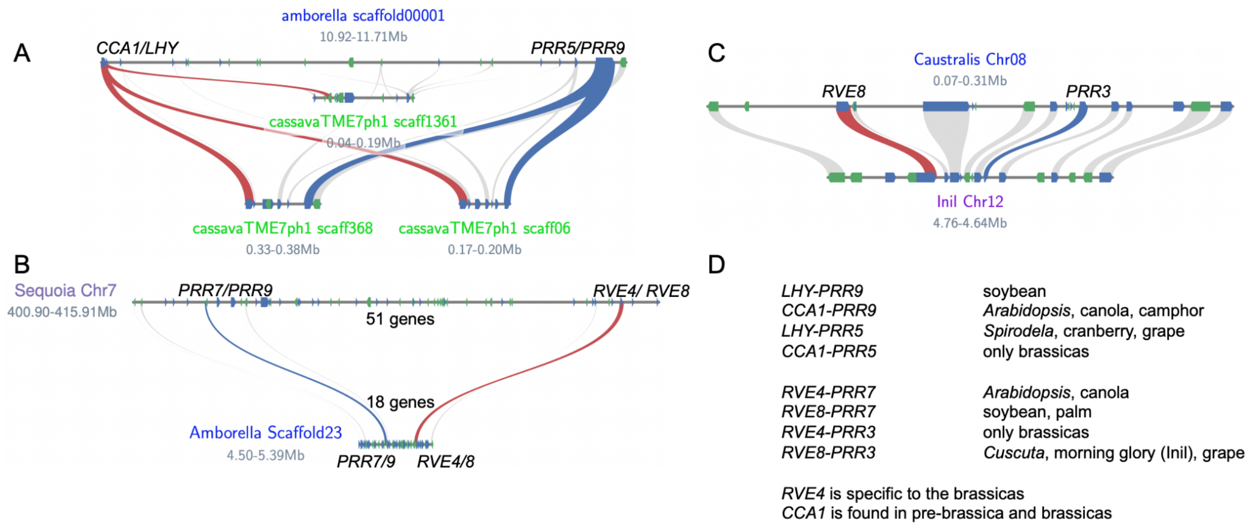

Supplemental Figure S7. Different *sMYB-PRR* combinations are found across plant genomes with distinct whole genome duplication (WGD) events. *LHY/CCA1/RVE* syntenic relationships (red), *PRR3/5/7/9* syntenic relationships (blue) and other syntenic genes (grey) for the entire syntenic block. Genes on the positive strand (blue) and negative strand (green). A) *Amborella* versus cassava (*Manihot esculenta*); B) Sequoia (*Sequoiadendron giganteum*) versus *Amborella*; C) Cuscuta (Caustralis; *Cuscuta australis*) versus Inil (*Ipomoea nil*) D) Eight different *sMYB-PRR* combinations and genome examples for each; this is not meant to be an exhaustive list.

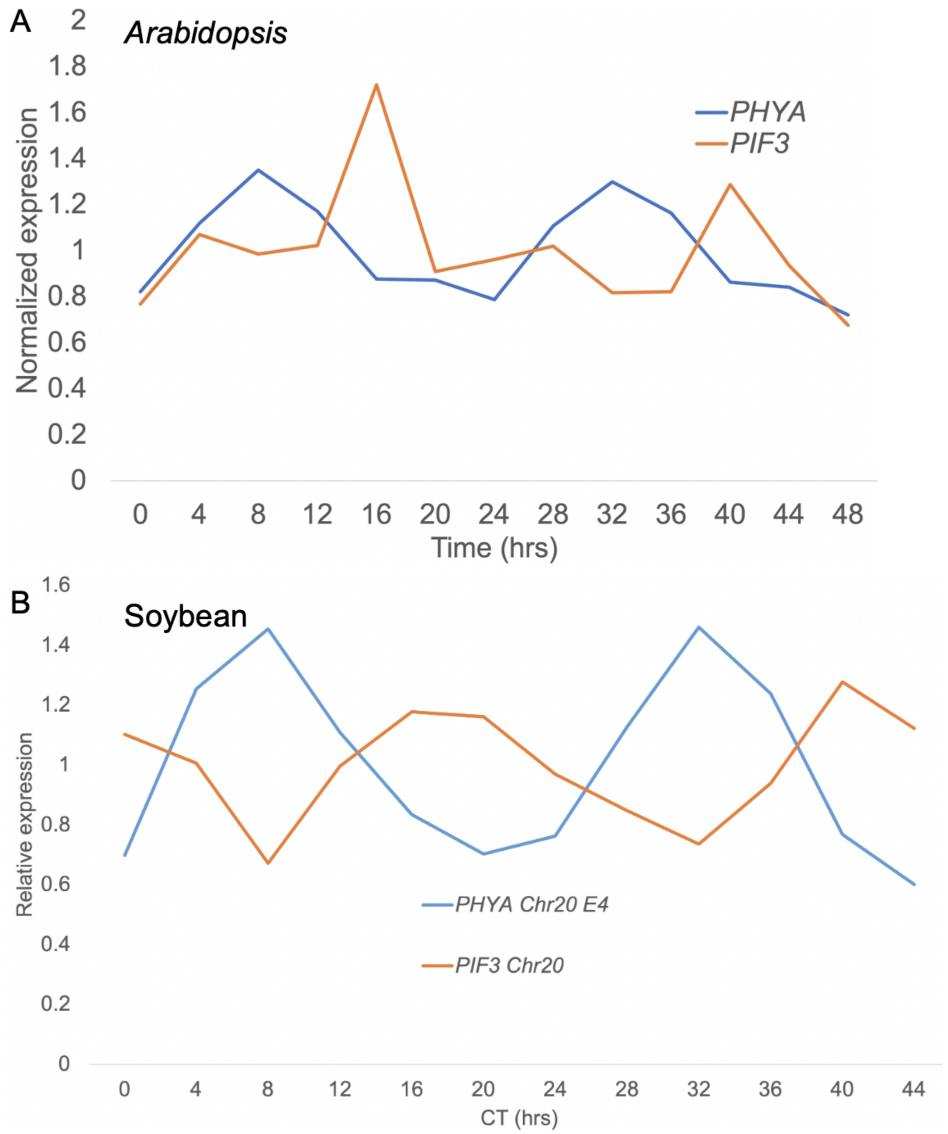

Supplemental Figure S8. *PIF3-PHYA* are expressed at distinct times of day in *Arabidopsis* and soybean. A) *Arabidopsis PHYA* (blue) shows peak expression at ZT11, while *PIF3* (orange) has peak expression ZT16. B) One syntenic pair of the four *PIF3-PHYA* linkages in soybean robustly cycles under circadian conditions with *PHYA* (blue) peaking at CT8 and *PIF3* (orange) peaking at CT17. ZT; Zeitgeber Time. CT; Circadian Time.

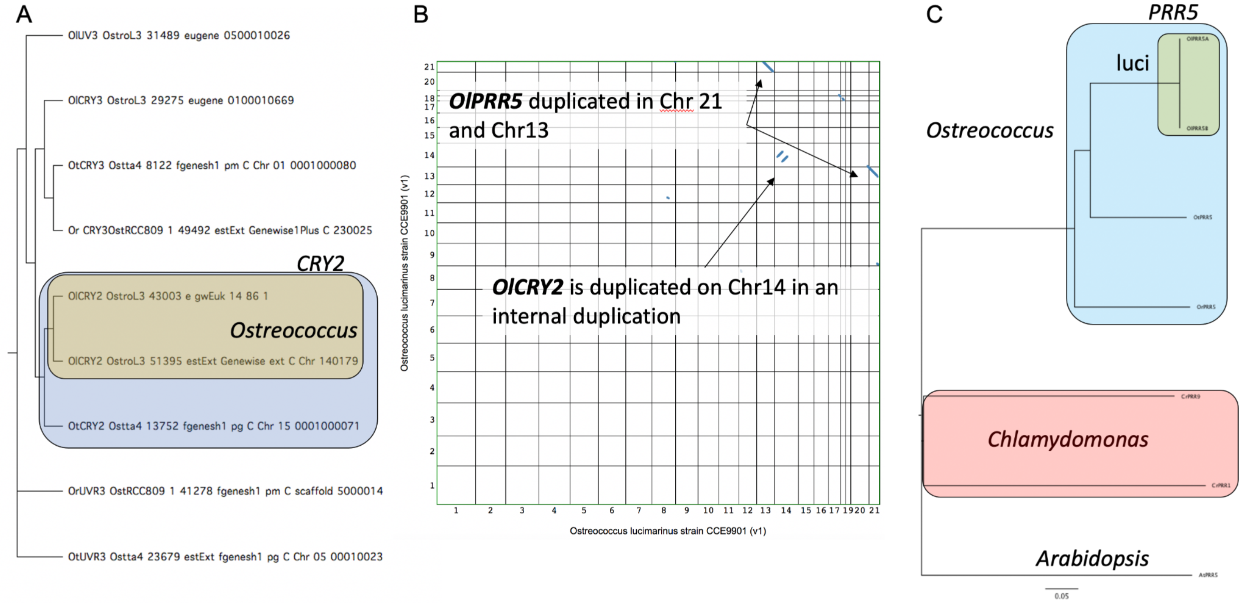

Supplemental Figure S9. Circadian clock and light signaling genes are duplicated in Ostreococcus. A) *CRYPTOCHROME* (*CRY/UVR*) family in Ostreococcus; all CRY2 genes (blue box) and duplicated *CRY2* genes in *O. lucimarinus* (brown box). B) Dotplot of *O. lucimarinus* (Ol) showing both the *CRY2* and *PRR5* duplications. C) *PRR* genes across *Arabidopsis*, *Chlamydomonas* (red box) and *Ostreococcus* (blue box). All of the *PRR* from *Ostreococcus* (*Ol*, *O. lucimarinus*; *Or*, *O. tauri*; and *Or*) and the duplicated *PRR5* in Ol (luci).

Supplemental Tables

Supplemental Table S1. ***Arabidopsis*** **circadian clock, light signaling and flowering time genes.**

Gene name	Gene ID		Gene name	Gene ID
sMYB family			light/flowering	
CCA1	AT2G46830		ARR3	At1g59940
LHY	AT1G01060		ARR4	At1g10470
RVE1	AT5G17300		bHLH69	At4g30980
RVE2	AT5G37260		bHLH92	At5g43650
RVE3	AT1G01520		CCR1	At4g39260
RVE4	AT5G02840		CCR2	At2g21660
RVE5	AT4G01280		COL2	At3g02380
RVE6	AT5G52660		COL9	At3g07650
RVE7	AT1G18330		COP1	At2g32950
RVE8	AT3G09600		CRB	At1g09340
			CRY1	At4g08920
PRR family			CRY2	At1g04400
PRR1	AT5G61380		DET1	At4g10180
PRR3	AT5G60100		EID1	At4g02440
PRR5	AT5G24470		FHY3	At3g22170
PRR7	AT5G02810		FIO1	At2g21070
PRR9	AT2G46790		FLC	At5g10140
			FT	At1g65480
ZTL			HYH	At3g17609
ZTL	AT5G57360		LIP1	At2g20860
LKP2	AT2G18915		LNK1	AT5G64170
FKF1	AT1G68050		LNK2	AT3G54500
			PHYA	At1g09570
GI	AT1G22770		PHYB	At2g18790
ELF3	AT2G25930		PIF3	At1g09530

ELF4	AT2G40080		PRMT5	At4g31120
PCL1/LUX	AT3G46640		SEC	At3g04240
LUX-like	AT5G59570		SFR6	At4g04920
			SPA1	At2g46340
TEJ	AT2G31870		SPY	At3g11540
TIC	AT3G22380		SRR1	At5g59560
CDF3	AT3G47500		STN7	At1g68830
CHE	AT5G08330			
CKB3	AT3G60250			
CKB4	AT2G44680			

Supplemental Table S2. Syntenic regions for core circadian clock genes in *Arabidopsis*.

Gene	Gene ID 1	Gene ID 1	Gene 2	Gene 1	Gene ID 1	Gene ID 1	Gene 2
	AT1G01030.1	AT2G46870.1			AT1G01100.1	AT4G00810.1	
	AT1G01050.1	AT2G46860.1			AT1G01110.2	AT4G00820.1	
LHY	AT1G01060.1	AT2G46830.1	CCA1		AT1G01160.1	AT4G00850.1	
	AT1G01120.1	AT2G46720.1			AT1G01170.1	AT4G00860.1	
	AT1G01190.1	AT2G46660.1			AT1G01225.1	AT4G00905.1	
	AT1G01240.1	AT2G46550.1			AT1G01340.2	AT4G01010.1	
	AT1G01260.1	AT2G46510.1			AT1G01350.1	AT4G01023.1	
	AT1G01340.2	AT2G46430.1			AT1G01360.1	AT4G01026.1	
	AT1G01380.1	AT2G46410.1			AT1G01380.1	AT4G01060.1	
	AT1G01440.1	AT2G46380.1			AT1G01420.1	AT4G01070.2	
	AT1G01453.1	AT2G46300.1			AT1G01430.1	AT4G01080.1	
					AT1G01440.1	AT4G01090.1	
	AT2G18650.1	AT5G57750.1			AT1G01453.1	AT4G01110.1	
	AT2G18730.1	AT5G57690.1			AT1G01460.1	AT4G01190.1	
	AT2G18750.1	AT5G57580.1		RVE3	AT1G01520.1	AT4G01280.1	RVE5
	AT2G18800.1	AT5G57550.1			AT1G01540.2	AT4G01330.1	
	AT2G18876.1	AT5G57410.3			AT1G01550.1	AT4G01360.1	
	AT2G18880.1	AT5G57380.1			AT1G01560.2	AT4G01370.1	
LKP2	AT2G18915.1	AT5G57360.2	ZTL				
	AT2G18960.1	AT5G57350.1			AT3G46350.1	AT5G59270.1	
	AT2G19160.1	AT5G57270.1			AT3G46440.1	AT5G59290.2	
	AT2G19230.1	AT5G57210.1			AT3G46460.1	AT5G59300.1	
					AT3G46520.1	AT5G59370.1	
	AT5G64960.1	AT5G10270.1			AT3G46580.1	AT5G59380.1	
	AT5G64990.1	AT5G10260.1			AT3G46590.1	AT5G59430.3	
	AT5G65010.1	AT5G10240.1			AT3G46600.1	AT5G59450.1	
	AT5G65020.1	AT5G10220.1			AT3G46613.1	AT5G59510.1	
	AT5G65030.1	AT5G10210.1			AT3G46620.1	AT5G59550.1	

MAF5	AT5G65080.1	AT5G10140.1	FLC	LUX	AT3G46640.3	AT5G59570.1	BOA
	AT5G65100.1	AT5G10120.1			AT3G46650.1	AT5G59580.1	
	AT5G65120.1	AT5G10110.1					
	AT5G65140.1	AT5G10100.1			AT3G09300.1	AT5G02100.1	
	AT5G65160.1	AT5G10090.1			AT3G09340.1	AT5G02170.2	
	AT5G65180.1	AT5G10060.1			AT3G09370.1	AT5G02320.1	
	AT5G65205.1	AT5G10050.1			AT3G09390.1	AT5G02380.1	
	AT5G65207.1	AT5G10040.1			AT3G09400.1	AT5G02400.1	
	AT5G65210.1	AT5G10030.1			AT3G09440.1	AT5G02500.1	
					AT3G09470.1	AT5G02502.1	
	AT5G08190.1	AT5G23090.1			AT3G09480.1	AT5G02570.1	
	AT5G08200.1	AT5G23130.1			AT3G09490.1	AT5G02590.1	
	AT5G08230.1	AT5G23150.1			AT3G09500.1	AT5G02610.1	
	AT5G08240.1	AT5G23160.1			AT3G09550.1	AT5G02620.1	
	AT5G08250.1	AT5G23190.1			AT3G09570.1	AT5G02630.1	
	AT5G08270.1	AT5G23200.1			AT3G09590.1	AT5G02730.1	
	AT5G08300.1	AT5G23250.1		RVE8	AT3G09600.1	AT5G02840.1	RVE4
CHE	AT5G08330.1	AT5G23280.1	TCP7		AT3G09630.1	AT5G02870.1	
	AT5G08335.1	AT5G23320.1			AT3G09670.1	AT5G02950.1	
	AT5G08340.2	AT5G23330.1			AT3G09680.1	AT5G02960.1	
	AT5G08350.1	AT5G23350.1			AT3G09690.1	AT5G02970.1	
	AT5G08360.1	AT5G23380.1			AT3G09700.1	AT5G03030.1	
	AT5G08390.1	AT5G23430.1			AT3G09710.1	AT5G03040.1	
	AT5G08410.1	AT5G23440.1			AT3G09760.1	AT5G03180.1	
	AT5G08430.1	AT5G23480.1			AT3G09770.1	AT5G03200.1	
	AT5G08440.1	AT5G23490.1			AT3G09790.1	AT5G03240.1	
	AT5G08500.1	AT5G23575.1			AT3G09810.1	AT5G03290.1	
	AT5G08520.1	AT5G23650.1			AT3G09820.1	AT5G03300.1	
					AT3G09840.1	AT5G03340.1	

Supplemental Table S3. Synonymous substitution (Ks) across *Arabidopsis* sMYB proteins. Coloring (from green to red) indicates the magnitude of the Ks value where green is a high number representing genes that are evolutionarily distant, and red indicates a low Ks value representing genes that are evolutionarily close.

Gene name	Gene ID	CCA1	LHY	RVE1	RVE2	RVE3	RVE4	RVE5	RVE6	RVE7	RVE8
		AT2G46830	AT1G01060	AT5G17300	AT5G37260	AT1G01520	AT5G02840	AT4G01280	AT5G52660	AT1G18330	AT3G09600
CCA1	AT2G46830		2.8531	3.2959	3.8734	3.8241	3.8795	3.8421	3.6285	3.9743	2.1194
LHY	AT1G01060	2.8531		3.9771	3.8654	3.8257	4.2384	3.9251	3.9332	3.9372	3.8884
RVE1	AT5G17300	3.2959	3.9771		3.9174	1.8631	5.648	2.3697	2.3213	4.0707	4.3232
RVE2	AT5G37260	3.8734	3.8654	3.9174		3.8757	2.2745	3.495	3.8795	3.742	2.4353
RVE3	AT1G01520	3.8241	3.8257	1.8631	3.8757		3.8886	1.2433	3.9338	3.8388	3.8348
RVE4	AT5G02840	3.8795	4.2384	5.648	2.2745	3.8886		2.1927	3.9253	3.92	0.8413
RVE5	AT4G01280	3.8421	3.9251	2.3697	3.495	1.2433	2.1927		3.9399	2.0931	1.9066
RVE6	AT5G52660	3.6285	3.9332	2.3213	3.8795	3.9338	3.9253	3.9399		3.9268	4.6156
RVE7	AT1G18330	3.9743	3.9372	4.0707	3.742	3.8388	3.92	2.0931	3.9268		3.9438
RVE8	AT3G09600	2.1194	3.8884	4.3232	2.4353	3.8348	0.8413	1.9066	4.6156	3.9438	

Supplemental Table S4. Synonymous substitution (Ks) across *Arabidopsis* PRR proteins. Coloring (from green to red) indicates the magnitude of the Ks value where green is a high number representing genes that are evolutionarily distant, and red indicates a low Ks value representing genes that are evolutionarily close.

Gene name	Gene ID	PRR1	PRR3	PRR5	PRR7	PRR9
		AT5G61380	AT5G60100	AT5G24470	AT5G02810	AT2G46790
PRR1	AT5G61380		4.1647	4.2759	4.3028	4.2329
PRR3	AT5G60100	4.1647		4.2775	4.2402	2.641
PRR5	AT5G24470	4.2759	4.2775		4.381	2.328
PRR7	AT5G02810	4.3028	4.2402	4.381		2.5112
PRR9	AT2G46790	4.2329	2.641	2.328	2.5112	

Supplemental Table S5. Genetic linkages between *CCA1/LHY-PRR5/9* and *RVE4/8-PRR3/7* from PLAZA dicot 4.5. For each species the number of genetic linkages is presented.

Abbreviation	Common Name	TaxID	PubMedID	LHY/CCA1-PRR5/9	RVE4/8-PRR3/7	Total linkages
ach	*Actinidia chinensis*	3625	24136039	0	0	0
Ahy	*Amaranthus hypochondriacus*	NA	NA	1	2	3
aip	*Arachis ipaensis*	130453	26901068	0	1	1
aly	*Arabidopsis lyrata*	59689	26382944	1	1	2
ath	*Arabidopsis thaliana*	3702	27862469	1	1	2
atr	*Amborella trichopoda*	13333	24357323	1	1	2
bol	*Brassica oleracea*	109376	24852848	1	0	1
bra	*Brassica rapa*	3711	21873998	0	1	1
bvu	*Beta vulgaris*	161934	24352233	1	0	1
can	*Capsicum annuum*	4072	24441736	0	0	0
car	*Cicer arietinum*	3827	23354103	1	1	2
ccaj	*Cajanus cajan*	3821	22057054	0	0	0
ccan	*Coffea canephora*	49390	25190796	1	1	2
ccl	*Citrus clementina*	85681	24908277	1	1	2
cla	*Citrullus lanatus*	3654	23179023	0	0	0
cme	*Cucumis melo*	3656	22753475	0	0	0
col	*Corchorus olitorius*	93759	28134914	1	0	1
cpa	*Carica papaya*	3649	18432245	0	0	0
cqu	*Chenopodium quinoa*	63459	28178233	2	0	2
cre	*Chlamydomonas reinhardtii*	3055	17932292	0	0	0
cru	*Capsella rubella*	81985	23749190	1	1	2
csa	*Cucumis sativus L.*	3659	NA	0	0	0
dca	*Daucus carota*	4039	27158781	0	0	0
egr	*Eucalyptus grandis*	71139	24919147	0	0	0
egut	*Erythranthe guttata*	4155	24225854	0	1	1

fve	*Fragaria vesca*	57918	21186353	0	1	1
gma	*Glycine max*	3847	20075913	4	2	6
gra	*Gossypium raimondii*	29730	22922876	1	1	2
hbr	*Hevea brasiliensis*	3981	27255837	1	2	3
mco	*Micromonas commoda*	296587	19359590	0	0	0
mdo	*Malus domestica*	3750	20802477	0	0	0
mes	*Manihot esculenta*	3983	22523606	0	0	0
mpo	*Marchantia polymorpha*	3197	28985561	0	0	0
mtr	*Medicago truncatula*	3880	22089132	1	1	2
nnu	*Nelumbo nucifera*	4432	23663246	0	1	1
osa	*Oryza sativa ssp. japonica*	39947	16100779	0	0	0
pab	*Picea abies*	3329	23698360	0	0	0
pax	*Petunia axillaris*	33119	27255838	0	0	0
pbr	*Pyrus bretschneideri*	225117	23149293	0	1	1
ppa	*Physcomitrella patens*	3218	18079367	0	0	0
ppe	*Prunus persica*	3760	23525075	0	1	1
ptr	*Populus trichocarpa*	3694	16973872	2	0	2
rco	*Ricinus communis*	3988	20729833	0	1	1
sly	*Solanum lycopersicum*	4081	22660326	1	0	1
smo	*Selaginella moellendorffii*	88036	21551031	0	0	0
spa	*Schrenkiella parvula*	98039	21822265	1	1	2
stu	*Solanum tuberosum*	4113	21743474	0	1	1
tca	*Theobroma cacao*	3641	21186351	1	1	2
tha	*Tarenaya hassleriana*	28532	23983221	1	2	3
tpr	*Trifolium pratense*	57577	26617401	1	0	1
ugi	*Utricularia gibba*	13748	23665961	0	0	0
vra	*Vigna radiata var. radiata*	157791	25384727	1	1	2
vvi	*Vitis vinifera*	29760	17721507	1	1	2
zju	*Ziziphus jujuba*	326968	25350882	0	0	0

zma	*Zea mays*	4577	19965430	0	0	0

Supplemental Table S6. Number of syntenic blocks and genetic linkages between *CCA1/LHY-PRR5/9*, *RVE4/8-PRR3/7* and *PIF3-PHYA*. The number under each individual gene pair is the number of syntenic blocks found per species. The number under the genetic linkages represents the number of species sharing genetic linkages with that species.

species	Clade	Order	*CCA1/LHY*	*RVE4/8*	*PRR3/7*	*PRR5/9*	*PHYA*	*PIF3*	*RVE4/8-PRR3/7*	*LHY/CCA1-PRR5/9*	*PIF3-PHYA*
Amborella trichopoda	Basal-Angiosperm	Amborellales	1	1	1	2	1	1	23	29	42
Nymphaea tetragona	Basal-Angiosperm	Nymphaeales	1	0	2	2	1	1	0	2	41
Nelumbo nucifera	Basal-Eudicots	Proteales	1	1	2	2	0	2	46	50	0
Macleaya cordata	Basal-Eudicots	Ranunculales	2	1	1	1	1	1	44	6	72
Papaver somniferum	Basal-Eudicots	Ranunculales	2	2	1	2	2	2	11	50	108
Aquilegia coerulea	Basal-Eudicots	Ranunculales	1	1	2	0	1	1	24	0	1
Vitis vinifera	Basal-Eudicots	Vitales	1	1	4	2	1	2	22	56	72
Cinnamomum micranthum	Magnoliids	Magnoliales	2	0	2	3	1	2	0	59	64
Persea americana	Magnoliids	Magnoliales	2	0	2	3	1	2	0	40	0
Liriodendron chinense	Magnoliids	Magnoliales	3	0	4	2	1	2	1	35	55
Spirodela polyrhiza	Monocots	Alismatales	2	1	1	1	2	2	0	2	0
Zostera marina	Monocots	Alismatales	0	0	0	0	0	2	0	0	0
Elaeis guineensis	Monocots	Arecales	1	1	3	0	1	4	50	1	41
Phoenix dactylifera	Monocots	Arecales	1	1	2	0	1	2	39	0	0
Asparagus officinalis	Monocots	Asparagales	1	1	1	0	1	5	17	0	31
Apostasia shenzhenica	Monocots	Asparagales	1	1	2	0	1	1	6	0	0
Phalaenopsis equestris	Monocots	Asparagales	1	0	1	0	1	0	0	0	0
Xerophyta viscosa	Monocots	Pandanales	0	1	3	1	1	3	0	0	2
Ananas comosus	Monocots	Poales	1	1	2	0	1	2	47	0	59
Brachypodium distachyon	Monocots	Poales	0	0	2	0	0	2	0	0	0
Echinochloa crus-galli	Monocots	Poales	0	0	4	0	0	6	0	0	0
Hordeum vulgare	Monocots	Poales	0	0	2	0	0	1	0	0	0
leersia perrieri	Monocots	Poales	0	0	3	0	0	2	0	0	0

Oropetium thomaeum	Monocots	Poales	0	0	3	0	0	2	0	0	0
Oryza glaberrima	Monocots	Poales	0	0	2	0	0	1	0	0	0
Oryza punctata	Monocots	Poales	0	0	3	0	0	2	0	0	0
Oryza rufipogon	Monocots	Poales	0	0	3	0	0	2	0	0	0
Oryza sativa	Monocots	Poales	0	0	2	0	0	1	0	0	0
Saccharum officinarum	Monocots	Poales	0	0	3	1	0	1	0	0	0
Setaria italica	Monocots	Poales	0	0	2	0	0	2	0	0	0
Setaria viridis	Monocots	Poales	0	0	2	0	0	2	0	0	0
Sorghum bicolor	Monocots	Poales	0	0	1	0	0	2	0	0	0
Triticum turgidum	Monocots	Poales	0	0	4	0	0	2	0	0	0
Zea mays	Monocots	Poales	0	0	3	0	1	3	0	0	0
Musa acuminata	Monocots	Zingiberales	0	1	4	0	2	2	0	0	0
Daucus carota	Super-Asterids	Apiales	1	1	3	3	2	1	0	0	71
Helianthus annuus	Super-Asterids	Asterales	0	1	7	1	0	0	0	0	0
Lactuca sativa	Super-Asterids	Asterales	1	1	3	1	1	0	53	0	0
Amaranthus hypochondriacus	Super-Asterids	Caryophyllales	2	1	2	2	2	2	46	58	0
Beta vulgaris	Super-Asterids	Caryophyllales	1	1	1	2	1	1	0	56	0
Chenopodium quinoa	Super-Asterids	Caryophyllales	2	1	3	4	2	2	7	59	0
Actinidia chinensis	Super-Asterids	Ericales	1	2	5	3	3	3	0	0	131
Actinidia eriantha	Super-Asterids	Ericales	2	3	6	3	3	4	1	1	85
Coffea canephora	Super-Asterids	Gentianales	1	1	2	1	1	1	27	0	0
Olea europaea	Super-Asterids	Lamiales	1	2	4	3	1	3	0	0	0
Sesamum indicum	Super-Asterids	Lamiales	1	1	1	2	2	1	55	0	76
Mimulus guttatus	Super-Asterids	Lamiales	1	1	2	2	2	1	53	0	74
Malania oleifera	Super-Asterids	Santalales	1	0	1	1	1	1	0	0	70
Kalanchoe fedtschenkoi	Super-Asterids	Saxifragales	2	1	5	2	2	2	0	17	71
Cuscuta campestris	Super-Asterids	Solanales	1	2	8	2	2	0	52	0	0
Ipomoea nil	Super-Asterids	Solanales	0	1	3	3	1	2	41	0	0
Capsicum annuum	Super-Asterids	Solanales	1	1	4	2	0	1	1	33	0
Capsicum baccatum	Super-Asterids	Solanales	2	1	2	2	0	1	0	32	0
Capsicum chinense	Super-Asterids	Solanales	1	1	2	2	0	1	0	0	0

Petunia axillaris	Super-Asterids	Solanales	0	1	3	3	0	2	3	0	0
Solanum lycopersicum	Super-Asterids	Solanales	1	1	3	2	0	1	10	34	0
Solanum pennellii	Super-Asterids	Solanales	1	1	2	2	0	1	1	35	0
Solanum tuberosum	Super-Asterids	Solanales	1	1	0	2	0	1	0	30	0
Aethionema arabicum	Super-Rosids	Brassicales	2	2	2	2	1	3	46	51	0
Arabidopsis lyrata	Super-Rosids	Brassicales	2	2	4	2	1	6	49	50	67
Arabidopsis thaliana	Super-Rosids	Brassicales	2	2	2	3	1	4	51	52	67
Arabis alpina	Super-Rosids	Brassicales	0	2	1	0	0	5	0	0	1
Boechera stricta	Super-Rosids	Brassicales	2	2	2	2	1	4	45	50	66
Brassica napus	Super-Rosids	Brassicales	3	10	7	9	4	15	50	48	61
Brassica oleracea	Super-Rosids	Brassicales	4	5	3	4	2	8	45	50	84
Brassica rapa	Super-Rosids	Brassicales	4	4	3	3	2	8	42	0	100
Camelina sativa	Super-Rosids	Brassicales	5	8	6	6	3	10	57	51	194
Capsella rubella	Super-Rosids	Brassicales	2	2	3	2	1	4	51	50	67
Lepidium meyenii	Super-Rosids	Brassicales	6	8	9	8	4	12	4	59	254
Schrenkiella parvula	Super-Rosids	Brassicales	1	1	2	2	1	5	0	50	65
Thellungiella halophila	Super-Rosids	Brassicales	1	2	5	2	1	3	50	1	66
Thellungiella salsuginea	Super-Rosids	Brassicales	1	2	2	2	1	3	45	1	66
Carica papaya	Super-Rosids	Brassicales	1	1	2	2	1	1	0	0	0
Cleome gynandra	Super-Rosids	Brassicales	3	1	1	4	0	3	0	50	0
Tarenaya hassleriana	Super-Rosids	Brassicales	5	2	6	4	2	6	53	25	62
Begonia fuchsioides	Super-Rosids	Cucurbitales	7	1	2	7	2	2	0	30	0
Citrullus lanatus	Super-Rosids	Cucurbitales	1	1	1	1	0	1	0	0	0
Cucumis melo	Super-Rosids	Cucurbitales	1	1	1	1	0	1	0	1	0
Cucumis sativus	Super-Rosids	Cucurbitales	1	1	1	1	0	1	0	0	0
Cucurbita maxima	Super-Rosids	Cucurbitales	2	0	2	3	0	2	0	0	0
Datisca glomerata	Super-Rosids	Cucurbitales	1	1	2	1	0	1	50	56	0
Ammopiptanthus nanus	Super-Rosids	Fabales	0	1	2	3	1	2	50	1	25
Arachis duranensis	Super-Rosids	Fabales	2	2	2	2	3	4	0	59	166
Cajanus cajan	Super-Rosids	Fabales	1	1	1	2	2	4	0	57	148
Cicer arietinum	Super-Rosids	Fabales	1	0	2	2	2	2	0	55	93

Glycine max	Super-Rosids	Fabales	4	4	4	6	4	8	53	61	337
Lotus japonicus	Super-Rosids	Fabales	1	1	1	8	1	3	51	52	75
Lupinus angustifolius	Super-Rosids	Fabales	3	1	2	3	3	7	0	55	181
Medicago truncatula	Super-Rosids	Fabales	1	1	2	3	1	3	55	51	92
Phaseolus vulgaris	Super-Rosids	Fabales	3	2	3	3	2	3	53	62	117
Trifolium pratense	Super-Rosids	Fabales	1	1	1	2	1	3	0	54	53
Vigna angularis	Super-Rosids	Fabales	2	2	3	3	2	4	31	51	180
Vigna radiata	Super-Rosids	Fabales	2	2	4	2	2	3	40	47	165
Betula pendula	Super-Rosids	Fagales	1	1	2	3	1	2	0	56	22
Casuarina glauca	Super-Rosids	Fagales	1	1	2	2	1	2	48	57	57
Quercus robur	Super-Rosids	Fagales	2	1	3	1	1	3	52	5	7
Carya illinoinensis	Super-Rosids	Fagales	1	0	3	2	0	3	0	0	0
Manihot esculenta	Super-Rosids	Malpighiales	2	1	5	3	1	3	16	58	78
Ricinus communis	Super-Rosids	Malpighiales	1	1	4	2	1	2	54	53	76
Linum usitatissimum	Super-Rosids	Malpighiales	5	2	2	6	0	2	50	7	0
Populus trichocarpa	Super-Rosids	Malpighiales	4	2	2	4	1	3	0	60	77
Durio zibethinus	Super-Rosids	Malvales	2	2	5	4	0	4	57	0	0
Gossypium barbadense	Super-Rosids	Malvales	5	2	8	9	0	6	47	58	2
Gossypium hirsutum	Super-Rosids	Malvales	6	2	8	8	0	6	47	58	2
Gossypium raimondii	Super-Rosids	Malvales	3	1	5	4	0	3	46	59	0
Theobroma cacao	Super-Rosids	Malvales	1	1	3	2	1	2	52	58	78
Punica granatum	Super-Rosids	Myrtales	1	1	2	2	2	3	49	0	74
Eucalyptus grandis	Super-Rosids	Myrtales	1	1	2	1	1	3	0	0	3
Trema orientale	Super-Rosids	Rosales	1	1	3	2	1	2	51	54	71
Morus notabilis	Super-Rosids	Rosales	1	1	5	2	1	2	50	55	75
Ziziphus jujuba	Super-Rosids	Rosales	1	0	3	2	3	2	0	2	79
Dryas drummondii	Super-Rosids	Rosales	1	1	2	2	1	1	53	51	75
Fragaria vesca	Super-Rosids	Rosales	2	1	1	2	1	1	51	60	61
Malus domestica	Super-Rosids	Rosales	2	1	5	3	2	3	57	54	149
Prunus mume	Super-Rosids	Rosales	1	1	1	1	1	1	53	0	74
Prunus persica	Super-Rosids	Rosales	2	1	1	2	1	1	52	55	76

Pyrus x bretschneideri	Super-Rosids	Rosales	2	1	4	2	2	3	54	1	74
Rosa chinensis	Super-Rosids	Rosales	1	1	2	2	1	2	50	58	73
Rubus occidentalis	Super-Rosids	Rosales	1	1	2	2	1	1	53	1	76
Parasponia andersonii	Super-Rosids	Rosales	1	1	3	2	1	2	0	32	75
Citrus maxima	Super-Rosids	Sapindales	1	0	3	2	1	2	0	58	58
Citrus sinensis	Super-Rosids	Sapindales	1	1	5	1	1	2	43	59	52
Xanthoceras sorbifolium	Super-Rosids	Sapindales	1	0	2	2	1	2	0	58	75

Supplemental Table S7. Summary of syntenic blocks across 123 plant genome assemblies for circadian genes.

	CCA1/LHY	*RVE4/8*	*PRR3/7*	*PRR5/9*	*PHYA*	*PIF3*
Family name	3634	4022	1555	1018	6934	851
Syntenic blocks (#)	182	152	345	264	129	330
Most syntenic blocks (#)	7	10	9	9	4	15
Fewest syntenic blocks (#)	0	0	0	0	0	0
Median syntenic blocks (#)	1	1	2	2	1	2
Average syntenic blocks (#)	1.5	1.2	2.8	2.1	1	2.7
Zero syntenic blocks	23	28	2	24	37	4
Total genomes tested (#)	123	123	123	123	123	123
missing (%)	18.70%	22.80%	1.60%	19.50%	30.10%	3.30%
present (%)	81.30%	77.20%	98.40%	80.50%	69.90%	96.70%

References

Adachi N, Lieber MR (2002) Bidirectional gene organization: a common architectural feature of the human genome. Cell **109**: 807–809

Alabadí D, Yanovsky MJ, Más P, Harmer SL, Kay SA (2002) Critical Role for CCA1 and LHY in Maintaining Circadian Rhythmicity in Arabidopsis. Current Biology **12**: 757–761

Bharadwaj R, Kumar SR, Sharma A, Sathishkumar R (2021) Plant Metabolic Gene Clusters: Evolution, Organization, and Their Applications in Synthetic Biology. Front Plant Sci **12**: 697318

Chaudhury A, Okada K, Raikhel NV, Shinozaki K, Sundaresan V V (1999) A weed reaches new heights down under. Plant Cell **11**: 1817–1826

Cheng F, Wu J, Cai X, Liang J, Freeling M, Wang X (2018) Gene retention, fractionation and subgenome differences in polyploid plants. Nat Plants **4**: 258–268

Chen W-H, de Meaux J, Lercher MJ (2010) Co-expression of neighbouring genes in Arabidopsis: separating chromatin effects from direct interactions. BMC Genomics **11**: 178

Condamine FL, Silvestro D, Koppelhus EB, Antonelli A (2020) The rise of angiosperms pushed conifers to decline during global cooling. Proc Natl Acad Sci U S A **117**: 28867–28875

Dávila López M, Martínez Guerra JJ, Samuelsson T (2010) Analysis of gene order conservation in eukaryotes identifies transcriptionally and functionally linked genes. PLoS One **5**: e10654

Dodd AN, Salathia N, Hall A, Kévei E, Tóth R, Nagy F, Hibberd JM, Millar AJ, Webb AAR (2005) Plant circadian clocks increase photosynthesis, growth, survival, and competitive advantage. Science **309**: 630–633

Farinas B, Mas P (2011) Functional implication of the MYB transcription factor RVE8/LCL5 in the circadian control of histone acetylation. Plant J **66**: 318–329

Farré EM, Harmer SL, Harmon FG, Yanovsky MJ, Kay SA (2005) Overlapping and distinct roles of PRR7 and PRR9 in the Arabidopsis circadian clock. Curr Biol **15**: 47–54

Fawcett JA, Maere S, Van de Peer Y (2009) Plants with double genomes might have had a better chance to survive the Cretaceous–Tertiary extinction event. Proc Natl Acad Sci U S A **106**: 5737–5742

Foflonker F, Blaby-Haas CE (2021) Colocality to Cofunctionality: Eukaryotic Gene Neighborhoods as a Resource for Function Discovery. Mol Biol Evol **38**: 650–662

Freeling M (2017) Picking up the ball at the K/pg boundary: The distribution of ancient polyploidies in the plant phylogenetic tree as a spandrel of asexuality with occasional sex. Plant Cell **29**: 202–206

Friedman J, Barrett SCH (2009) Wind of change: new insights on the ecology and evolution of

pollination and mating in wind-pollinated plants. Ann Bot **103**: 1515–1527

Gray JA, Shalit-Kaneh A, Chu DN, Hsu PY, Harmer SL (2017) The REVEILLE Clock Genes Inhibit Growth of Juvenile and Adult Plants by Control of Cell Size. Plant Physiol **173**: 2308–2322

Green RM, Tobin EM (1999) Loss of the circadian clock-associated protein 1 in Arabidopsis results in altered clock-regulated gene expression. Proc Natl Acad Sci U S A **96**: 4176–4179

Hsu PY, Devisetty UK, Harmer SL (2013) Accurate timekeeping is controlled by a cycling activator in Arabidopsis. Elife **2**: e00473

Hurst LD, Pál C, Lercher MJ (2004) The evolutionary dynamics of eukaryotic gene order. Nat Rev Genet **5**: 299–310

Kautsar SA, Suarez Duran HG, Blin K, Osbourn A, Medema MH (2017) plantiSMASH: automated identification, annotation and expression analysis of plant biosynthetic gene clusters. Nucleic Acids Res **45**: W55–W63

Kuno N, Møller SG, Shinomura T, Xu X, Chua N-H, Furuya M (2003) The novel MYB protein EARLY-PHYTOCHROME-RESPONSIVE1 is a component of a slave circadian oscillator in Arabidopsis. Plant Cell **15**: 2476–2488

Lee JM, Sonnhammer ELL (2003) Genomic gene clustering analysis of pathways in eukaryotes. Genome Res **13**: 875–882

Linder HP, Lehmann CER, Archibald S, Osborne CP, Richardson DM (2018) Global grass (Poaceae) success underpinned by traits facilitating colonization, persistence and habitat transformation. Biol Rev Camb Philos Soc **93**: 1125–1144

Lu SX, Knowles SM, Andronis C, Ong MS, Tobin EM (2009) CIRCADIAN CLOCK ASSOCIATED1 and LATE ELONGATED HYPOCOTYL Function Synergistically in the Circadian Clock of Arabidopsis. Plant Physiology **150**: 834–843

Marcet-Houben M, Gabaldón T (2020) EvolClust: automated inference of evolutionary conserved gene clusters in eukaryotes. Bioinformatics **36**: 1265–1266

Marcet-Houben M, Gabaldón T (2019) Evolutionary and functional patterns of shared gene neighbourhood in fungi. Nature Microbiology **4**: 2383–2392

Matsushika A, Makino S, Kojima M, Mizuno T (2000) Circadian waves of expression of the APRR1/TOC1 family of pseudo-response regulators in Arabidopsis thaliana: insight into the plant circadian clock. Plant Cell Physiol **41**: 1002–1012

Michael TP, Mockler TC, Breton G, McEntee C, Byer A, Trout JD, Hazen SP, Shen R, Priest HD, Sullivan CM, et al (2008) Network Discovery Pipeline Elucidates Conserved Time-of-Day–Specific cis-Regulatory Modules. PLoS Genet **4**: e14

Michael TP, Salomé PA, Yu HJ, Spencer TR, Sharp EL, McPeek MA, Alonso JM, Ecker JR, McClung CR (2003) Enhanced fitness conferred by naturally occurring variation in the circadian clock. Science **302**: 1049–1053

Michalak P (2008) Coexpression, coregulation, and cofunctionality of neighboring genes in eukaryotic genomes. Genomics **91**: 243–248

Millar AJ, Carré IA, Strayer CA, Chua NH, Kay SA (1995) Circadian clock mutants in Arabidopsis identified by luciferase imaging. Science **267**: 1161–1163

Mizoguchi T, Wheatley K, Hanzawa Y, Wright L, Mizoguchi M, Song HR, Carré IA, Coupland G (2002) LHY and CCA1 are partially redundant genes required to maintain circadian rhythms in Arabidopsis. Dev Cell **2**: 629–641

Nimmo HG, Laird J (2021) Arabidopsis thaliana PRR7 Provides Circadian Input to the CCA1 Promoter in Shoots but not Roots. Front Plant Sci **12**: 750367

Nützmann H-W, Doerr D, Ramírez-Colmenero A, Sotelo-Fonseca JE, Wegel E, Di Stefano M, Wingett SW, Fraser P, Hurst L, Fernandez-Valverde SL, et al (2020) Active and repressed biosynthetic gene clusters have spatially distinct chromosome states. Proc Natl Acad Sci U S A **117**: 13800–13809

Nützmann H-W, Scazzocchio C, Osbourn A (2018) Metabolic Gene Clusters in Eukaryotes. Annu Rev Genet **52**: 159–183

Oda A, Reeves PH, Tajima T, Nakagawa M, Kamada H, Coupland G, Mizoguchi T (2007) Isolation of novel gain- and loss-of-function alleles of the circadian clock gene LATE ELONGATED HYPOCOTYL (LHY) in Arabidopsis. Plant Biotechnology **24**: 457–465

Osbourn A (2010) Gene clusters for secondary metabolic pathways: an emerging theme in plant biology. Plant Physiol **154**: 531–535

Pál C, Hurst LD (2003) Evidence for co-evolution of gene order and recombination rate. Nat Genet **33**: 392–395

Rawat R, Schwartz J, Jones MA, Sairanen I, Cheng Y, Andersson CR, Zhao Y, Ljung K, Harmer SL (2009) REVEILLE1, a Myb-like transcription factor, integrates the circadian clock and auxin pathways. Proc Natl Acad Sci U S A **106**: 16883–16888

Rawat R, Takahashi N, Hsu PY, Jones MA, Schwartz J, Salemi MR, Phinney BS, Harmer SL (2011) REVEILLE8 and PSEUDO-REPONSE REGULATOR5 form a negative feedback loop within the Arabidopsis circadian clock. PLoS Genet **7**: e1001350

Regal PJ (1977) Ecology and evolution of flowering plant dominance. Science **196**: 622–629

Rocha EPC (2008) The organization of the bacterial genome. Annu Rev Genet **42**: 211–233

Salomé PA, McClung CR (2005) PSEUDO-RESPONSE REGULATOR 7 and 9 are partially redundant genes essential for the temperature responsiveness of the Arabidopsis circadian clock. Plant Cell **17**: 791–803

Salomé PA, Weigel D, McClung CR (2010) The role of the Arabidopsis morning loop components CCA1, LHY, PRR7, and PRR9 in temperature compensation. Plant Cell **22**: 3650–3661

Schaffer R, Ramsay N, Samach A, Corden S, Putterill J, Carré IA, Coupland G (1998) The late elongated hypocotyl mutation of Arabidopsis disrupts circadian rhythms and the

photoperiodic control of flowering. Cell **93**: 1219–1229

Shalit-Kaneh A, Kumimoto RW, Filkov V, Harmer SL (2018) Multiple feedback loops of the Arabidopsis circadian clock provide rhythmic robustness across environmental conditions. Proc Natl Acad Sci U S A **115**: 7147–7152

Soltis DE, Albert VA, Leebens-Mack J, Bell CD, Paterson AH, Zheng C, Sankoff D, Depamphilis CW, Wall PK, Soltis PS (2009) Polyploidy and angiosperm diversification. Am J Bot **96**: 336–348

Somers DE, Webb AA, Pearson M, Kay SA (1998) The short-period mutant, toc1-1, alters circadian clock regulation of multiple outputs throughout development in Arabidopsis thaliana. Development **125**: 485–494

Strayer C, Oyama T, Schultz TF, Raman R, Somers DE, Más P, Panda S, Kreps JA, Kay SA (2000) Cloning of the Arabidopsis clock gene TOC1, an autoregulatory response regulator homolog. Science **289**: 768–771

Trinklein ND, Aldred SF, Hartman SJ, Schroeder DI, Otillar RP, Myers RM (2004) An abundance of bidirectional promoters in the human genome. Genome Res **14**: 62–66

Vision TJ (2005) Gene order in plants: a slow but sure shuffle. New Phytol **168**: 51–60

Wang ZY, Tobin EM (1998) Constitutive expression of the CIRCADIAN CLOCK ASSOCIATED 1 (CCA1) gene disrupts circadian rhythms and suppresses its own expression. Cell **93**: 1207–1217

Williams EJB, Bowles DJ (2004) Coexpression of neighboring genes in the genome of Arabidopsis thaliana. Genome Res **14**: 1060–1067

Winter S, Jahn K, Wehner S, Kuchenbecker L, Marz M, Stoye J, Böcker S (2016) Finding approximate gene clusters with Gecko 3. Nucleic Acids Res **44**: 9600–9610

Zhang X, Chen Y, Wang Z-Y, Chen Z, Gu H, Qu L-J (2007) Constitutive expression of CIR1 (RVE2) affects several circadian-regulated processes and seed germination in Arabidopsis. The Plant Journal **51**: 512–525

Zhan S, Horrocks J, Lukens LN (2006) Islands of co-expressed neighbouring genes in Arabidopsis thaliana suggest higher-order chromosome domains. Plant J **45**: 347–357

Zhao M, Zhang B, Lisch D, Ma J (2017) Patterns and Consequences of Subgenome Differentiation Provide Insights into the Nature of Paleopolyploidy in Plants. Plant Cell **29**: 2974–2994

Zhao T, Schranz ME (2019) Network-based microsynteny analysis identifies major differences and genomic outliers in mammalian and angiosperm genomes. Proc Natl Acad Sci U S A **116**: 2165–2174
